# Characterizing replisome disassembly in human cells

**DOI:** 10.1016/j.isci.2024.110260

**Published:** 2024-06-12

**Authors:** Rebecca M. Jones, Joaquin Herrero Ruiz, Shaun Scaramuzza, Sarmi Nath, Chaoyu Liu, Marta Henklewska, Toyoaki Natsume, Robert G. Bristow, Francisco Romero, Masato T. Kanemaki, Agnieszka Gambus

**Affiliations:** 1 Institute of Cancer and Genomic Sciences, Birmingham Centre for Genome Biology, University of Birmingham, Birmingham, UK; 2 Department of Chromosome Science, National Institute of Genetics, Research Organization of Information and Systems, Mishima, Shizuoka, Japan; 3 Department of Genetics, The Graduate University for Advanced Studies (SOKENDAI), Mishima, Shizuoka, Japan; 4 Department of Microbiology, University of Seville, Seville, Spain; 5 Department of Biological Science, The University of Tokyo, Tokyo, Japan; 6 Cancer Research UK – Manchester Institute, Manchester Cancer Research Center, Manchester, UK

**Keywords:** biochemistry, genetics, cell biology

## Abstract

To ensure timely duplication of the entire eukaryotic genome, thousands of replication machineries (replisomes) act on genomic DNA at any time during S phase. In the final stages of this process, replisomes are unloaded from chromatin. Unloading is driven by polyubiquitylation of MCM7, a subunit of the terminated replicative helicase, and processed by p97/VCP segregase. Most of our knowledge of replication termination comes from model organisms, and little is known about how this process is executed and regulated in human somatic cells. Here we show that replisome disassembly in this system requires CUL2^LRR1^-driven MCM7 ubiquitylation, p97, and UBXN7 for unloading and provide evidence for “backup” mitotic replisome disassembly, demonstrating conservation of such mechanisms. Finally, we find that small-molecule inhibitors against Cullin ubiquitin ligases (CULi) and p97 (p97i) affect replisome unloading but also lead to induction of replication stress in cells, which limits their usefulness to specifically target replisome disassembly processes.

## Highlights

• CUL2^LRR1^ interacts with human replisome in S phase and is required for MCM7 ubiquitylation
• CUL2^LRR1^ and p97-UBXN7 are required for human replisome unloading in S phase
• Mitotic replisome disassembly pathway exists in human cells
• Cullin and p97 inhibitors cannot specifically target CMG unloading in human cells

## Graphical abstract

**Figure:**
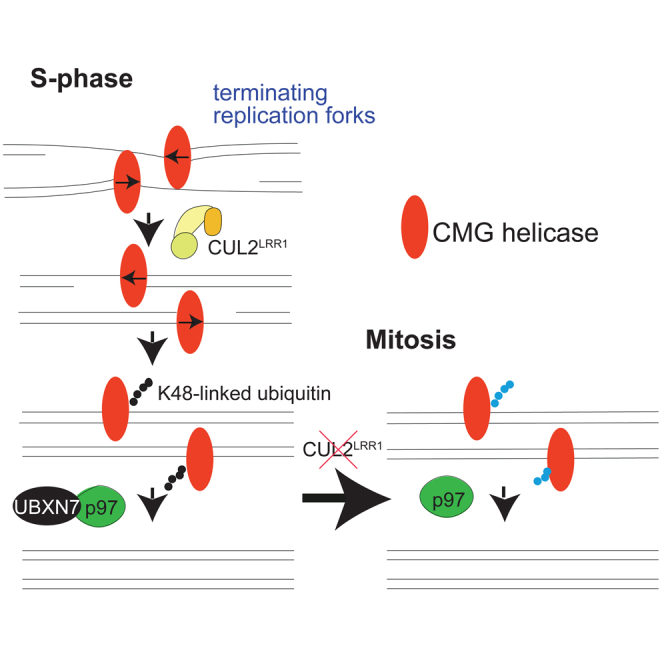

## Introduction

**Figure 1:**
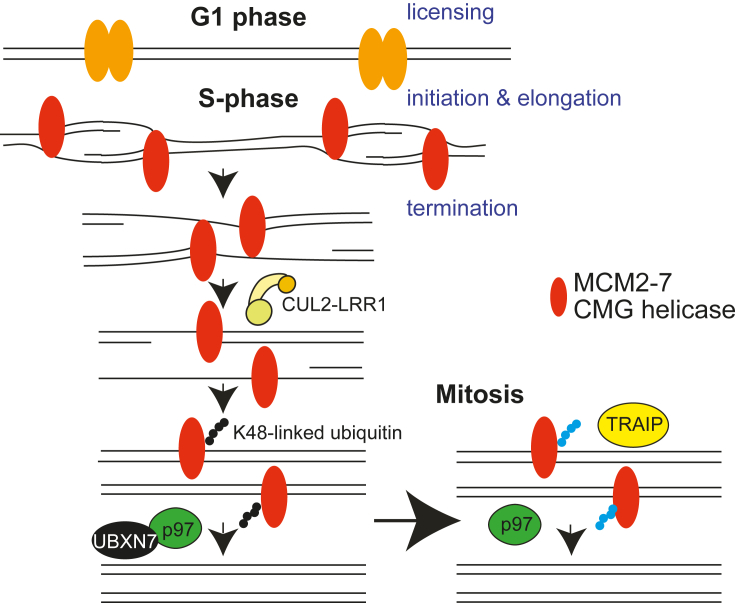
Schematic of replisome unloading during S-phase and mitosis

Cell division is the basis for the propagation of life and requires accurate duplication of all genetic information. The perfect execution of DNA replication is essential to maintain a stable genome and protect from diseases such as genetic disorders, cancer, and premature aging.^1^ Fundamental studies over the last 60 years have led to a step change in our understanding of DNA replication initiation and DNA synthesis,^2^ but, until the discovery of the first elements of the replisome disassembly mechanism in 2014,^3^^,^^4^ the termination stage of eukaryotic replication was mostly unexplored. DNA replication termination is triggered whenever two replication forks, coming from neighboring origins, meet each other and converge in a head-to-head orientation. During termination, the final fragments of DNA need to be replicated, the replisomes disassembled, and DNA intertwines resolved. A major difficulty when investigating replication termination is that the genomic position of replication fork termination and convergence is stochastic, occurring wherever two forks meet. Throughout S phase in eukaryotic cells, replication forks undergo initiation, elongation, and termination at different times; early replication forks will terminate before late replicating forks are initiated. Therefore, it is difficult to study the termination stage of DNA replication at the ensemble level. Consequently, replication termination has thus far been predominantly studied in reconstituted biochemical systems and model organisms that allow the termination of forks in a synchronized and controlled fashion. In the last ten years, biochemistry and cell biology research carried out using *Saccharomyces cerevisiae*, *Xenopus laevis* egg extracts, and *Caenorhabditis elegans* embryos has allowed us to elucidate the first model of the mechanism of eukaryotic DNA replication termination, with the unloading of the replication machinery (replisome disassembly) being the best understood stage of this process thus far (Figure 1).^3^^,^^4^^,^^5^^,^^6^^,^^7^^,^^8^^,^^9^^,^^10^^,^^11^^,^^12^^,^^13^^,^^14^^,^^15^^,^^16^

The eukaryotic replicative helicase is a complex formed of three components: CDC45, MCM2-7 hexamer, and GINS complex (SLD5, PSF1-3), altogether referred to as the CMG complex. Upon termination, MCM7 is specifically polyubiquitylated to promote the removal of the helicase from chromatin in a p97/VCP segregase-dependent manner. As the CMG helicase forms the organizing center of the replisome, its removal leads to disassembly of the entire replisome.^4^ Importantly, the main elements of this process are evolutionarily conserved in *S. cerevisiae*,^3^ *C. elegans*,^9^ and *X. laevis* egg extract.^4^ Following on from these initial findings, we and others went on to identify the CUL2^LRR1^ ubiquitin ligase (LRR1 is a substrate-specific receptor for the ligase) as the enzyme responsible for MCM7 ubiquitylation in S phase in higher eukaryotes.^9^^,^^14^ CUL2^LRR1^ can specifically recognize terminated helicases, as its substrate binding requires availability of an interaction surface on the CMG helicase, which is obscured by the single-stranded DNA (ssDNA) of the excluded lagging strand, protruding from CMG during unwinding at replication forks.^5^^,^^17^^,^^18^ Once MCM7 is ubiquitylated with K48-linked ubiquitin chains, it is recognized by the p97-UFD1-NPL4 complex with the help of the UBXN7 cofactor.^4^^,^^9^^,^^12^^,^^15^ Consequently, p97-driven unfolding of MCM7 leads to breaking of the CMG complex and its unloading from chromatin. When the LRR1-dependent mechanism fails, there is a backup pathway of replisome disassembly, activated in mitosis, and regulated by the TRAIP ubiquitin ligase.^6^^,^^9^^,^^11^ TRAIP ubiquitin ligase was also shown to act during S phase to unload replisomes that converge at inter-strand crosslinks (ICLs) to allow for their repair.^19^ Much of this mechanism of replisome disassembly has been also recently confirmed to be conserved in mouse embryonic stem cells where, unlike in human cells, TRAIP is not essential for cell viability.^10^ Although the basic elements of this process are conserved between fungi and metazoa, some of the factors and regulations involved differ. For example, in *S. cerevisiae*, Mcm7 is ubiquitylated during termination by SCF^Dia2^ and TRAIP ubiquitin ligase simply does not exist. Hence, the failure of unloading during S phase results in replisome persistence on chromatin until the next cell cycle.^3^

In higher eukaryotes, the process of replisome disassembly has been studied thus far mostly in embryonic systems as these models pose advantages that make them particularly useful for the study of DNA replication. However, it is known that embryonic cell cycles and DNA replication are regulated somewhat differently than in somatic cells. For example, the stabilization of CDT1 during DNA replication is not enough to induce re-replication in *Xenopus laevis* egg extract,^20^ as it is in human somatic cells^21^; while origin firing is regulated by both ataxia telangiectasia mutated (ATM) and ataxia telangiectasia and Rad3 related (ATR) in embryonic stem cells, but mainly by ATR in somatic cells.^22^ Moreover, embryonic systems are known to maintain fast cell cycles with non-existent or short gap phases, with reduced regulation of cell-cycle phase transition and accumulation of high levels of replication factors to sustain this fast proliferation rate. The consequences of dysregulating the process of replisome disassembly may therefore differ between the embryonic systems and somatic cells. Indeed, the first study focusing on this process in human MCF10A epithelial cells has suggested that short-term CRISPR-mediated LRR1 depletion leads to problems with replisome disassembly during S phase, resulting in the failure of S phase completion due to a reduction in the pool of available CDC45 and GINS required to fire late origins.^16^ Finally, earlier studies of human CUL2^LRR1^ showed that it is needed to regulate the cytoplasmic pool of CDK inhibitor p21 and regulate cell migration.^23^

Here, we have set out to explore the mechanism of replisome disassembly in human somatic cells. We aimed to set up assays to study this process in model cell lines, validate tools to study this process, and uncover similarities and differences between somatic and embryonic regulation of replisome disassembly. We found that all elements of this process, previously reported in higher eukaryotic model systems, are conserved in human cell lines: MCM7 is ubiquitylated with K48-linked ubiquitin chains on S phase chromatin; CUL2^LRR1^ interacts with replisome in S phase and is required for replisome disassembly during S phase; there is a mitotic backup pathway of replisome disassembly; and p97 is needed for unloading of replisomes both in S phase and in mitosis. We have found however that replication progression and replisome disassembly are much more tightly regulated in tissue culture somatic cells, compared with model systems used previously to study replisome disassembly. As a result, we find that the inhibitors commonly used as tools to study this process in model systems are less appropriate for use in somatic cells.

## Results

### Replisome unloading in human cell lines

To determine the mechanism of replisome unloading from chromatin in human cells, we first established methods to visualize unloading of the replicative helicase from the chromatin (Figure S1). Firstly, we observed the pattern of chromatin binding of the MCM7 subunit of MCM2-7 complex using fluorescence-activated cell sorting (FACS) on detergent-extracted HCT116 cells, where only chromatin-bound MCM7 signal is analyzed (Figure S1A). The MCM2-7 complex is initially loaded onto origins of replication throughout G1 stage of the cell cycle. In S phase, some of the MCM2-7 hexamers are activated to form CMGs and replisomes. During the process of DNA replication, the replisomes, as well as inactive MCM2-7 complexes (dormant origins), are unloaded as termination events occur.^24^^,^^25^ We have seen that the highest levels of chromatin-bound MCM7 are reached in G1 cells and that these levels decrease throughout S phase, with the majority of G2/M stage cells then having the lowest MCM7 intensities (Figure S1A). We can also visualize MCM2-7 complexes on chromatin by immunofluorescence of extracted cell nuclei, and with this we can detect cells with varying levels of MCM7 on chromatin in an asynchronous population (Figure S1B). To assign cell-cycle stage status to each cell, we used EdU incorporation into DNA to identify actively replicating cells; to identify G2 cells we utilized staining with CENPF/Mitosin—a large kinetochore-associated protein that is commonly used as a cell-cycle marker due to its homogeneous nuclear distribution in G2 and mitosis^26^; and to identify mitotic cells, we used staining with phospho-histone H3 S10 (pH3-S10)—a universal marker of chromosome condensation in eukaryotes.^27^ Cells, which are negative for EdU and CENPF signal, were classed as in G1. Cells that were positive for EdU and had increasing staining for CENPF were classed as early/mid/late S phase. Those negative for EdU with strong staining for CENPF were classed as G2. Finally, those with strong staining for phospho-histone H3 S10 (pH3-S10) and displaying chromatin condensation phenotype by DAPI staining were classed as mitotic. In both S phase and G2 cells, the level of MCM7 on chromatin was lower than that seen in G1 cells (Figure S1B). To further support this, U2OS cells were synchronized in early S phase with a double thymidine block (DTB) and then released into S phase, following which we analyzed the levels of chromatin-bound MCM7 through western blotting (Figure S1C-D) and immunofluorescence (Figure S1E). In both assays, we could detect the highest levels of MCM7 at the 0 h time point (early S phase), with levels dropping throughout S phase. In order to detect the active CMG complex specifically, we also analyzed levels of chromatin-bound CDC45 through western blotting (Figure S1C) and immunofluorescence (Figure S1F). As expected, we could only detect CDC45 associating with chromatin during S phase when DNA replication is taking place. With all this, we had now established methods for analyzing replisome disassembly in human cells.

### MCM7 is ubiquitylated on S phase chromatin with K48-linked ubiquitin chains

**Figure 2:**
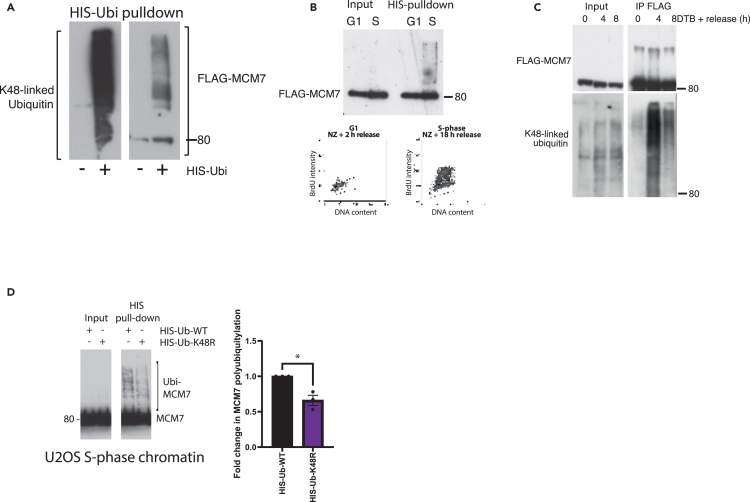
MCM7 is polyubiquitylated with K48-linked chains in S-phase (A) MCM7 is ubiquitylated on chromatin. U2OS cells expressing FLAG-MCM7 were optionally transfected with HIS-Ubi plasmid for 48 h. Cells from asynchronous population were extracted with CSK buffer, and HIS-tagged/ubiquitylated proteins were then isolated from chromatin samples under denaturing conditions and samples analyzed through western blotting with indicated antibodies. (B) MCM7 is ubiquitylated on chromatin during S-phase. As in (A) but cells were initially synchronized with nocodazole and released so as to gather cells from G1 and S-phase. FLAG-MCM7 signal was detected in HIS-Ubi pull-down samples and bromodeoxyuridine (BrdU) incorporation was determined by FACS analysis for G1 and S-phase samples. (C) MCM7 is ubiquitylated on chromatin in S-phase with K48-linked ubiquitin chains. U2OS cells expressing FLAG-MCM7 were synchronized with DTB and then released for the indicated time before cells were extracted with CSK buffer in denaturing conditions. FLAG-MCM7 was then isolated from chromatin with anti-FLAG beads and samples analyzed through western blotting with indicated antibodies. (D) U2OS cells were transfected with HIS-Ubi-WT or HIS-Ubi-K48R plasmid, synchronized with STB and then released for 6 h. HIS-tagged proteins were isolated using the HIS pull-down assay and samples analyzed by western blotting with the indicated antibodies. Levels of MCM7 ubiquitylation were quantified using ImageJ^29^ (*n* = 3) (*p* = 0.0424, Two-tailed paired t test); mean value +/− SEM.

Previous studies into the mechanism of replication termination revealed that MCM7 is ubiquitylated prior to replisome disassembly.^3^^,^^4^ We thus aimed to observe the ubiquitylation of MCM7 on chromatin. To this end, we established a U2OS cell line expressing FLAG-tagged MCM7 (FLAG-MCM7), as well as one expressing FLAG-MCM4, as a control. Expression levels of this exogenous FLAG-MCM7 are very close to those of the endogenous MCM7, and FLAG-MCM7 binds chromatin equally well (Figure S2A). Cells expressing FLAG-MCM7 also show a normal cell-cycle profile (Figure S2B) and display a similar profile of FLAG-MCM7 unloading from chromatin, compared to endogenous MCM7 (Figure S2C). Moreover, FLAG-MCM7 co-immunoprecipitates with the CMG helicase component GINS (Figure S2D). Importantly, the immunoblotting antibody signal is much cleaner for FLAG than endogenous MCM7 antibody, which allows us to better detect ubiquitylated forms of FLAG-MCM7. To analyze this ubiquitylated MCM7, we transfected cells with a HIS-tagged ubiquitin plasmid and performed a HIS pull-down to isolate all ubiquitylated proteins. MCM7 has been reported previously to be ubiquitylated on chromatin in human cells,^28^ and we could reproduce this observation (Figure 2A). To confirm that this ubiquitylation occurs in S phase, cells were synchronized in mitosis with nocodazole and released into G1 and then S phase. Using the HIS pull-down assay, we could clearly see that FLAG-MCM7 is indeed ubiquitylated specifically in S phase and not in the G1 stage of the cell cycle (Figure 2B). To further support this, we immunoprecipitated FLAG-MCM7 from chromatin of cells synchronized in S phase with release from DTB and could detect a ladder of bands over FLAG-MCM7 at the 4 h release time point (Figure 2C), suggesting that FLAG-MCM7 is ubiquitylated upon recovery of DNA synthesis (Figure S2E). Using an antibody specific to K48-linked ubiquitin chains, we could detect a strong signal over the size of FLAG-MCM7, supporting the model that MCM7 is ubiquitylated in S phase with K48-linked ubiquitin chains (Figure 2C). Finally, we transfected cells with either a wild type (WT) or K48R mutant of HIS-tagged ubiquitin and repeated the pull-down assay from S phase cells. Ubiquitylation of MCM7 on S phase chromatin was strongly diminished upon K48R ubiquitin mutant expression (Figure 2D).

### CUL2^LRR1^ interacts with the replisome on chromatin in S phase

**Figure 3:**
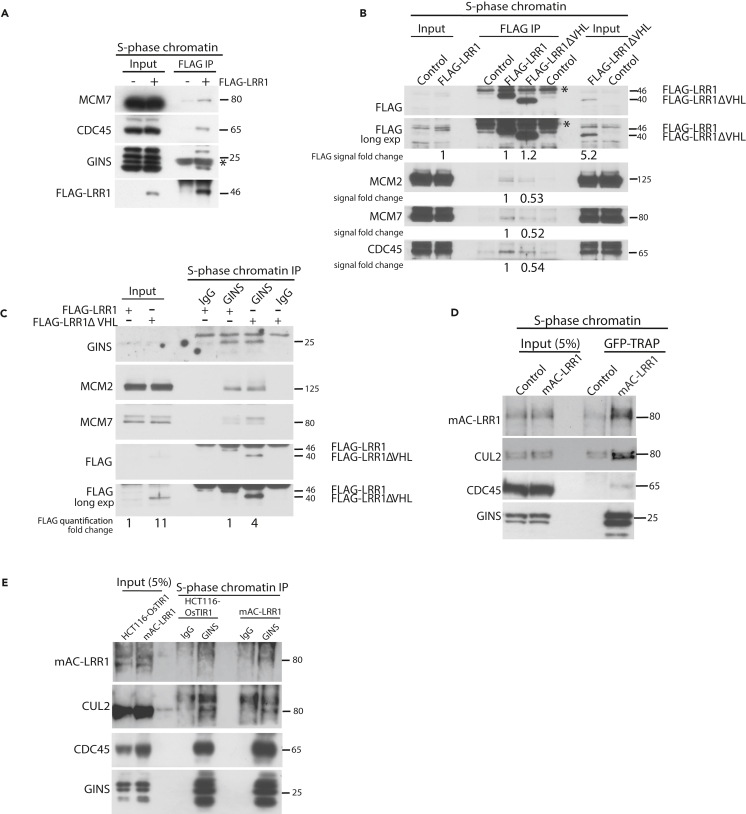
CUL2^LRR1^ interacts with the replisome during S-phase (A) LRR1 interacts with replisome components on chromatin during S-phase. Chromatin extracts of FLAG-LRR1 expressing cells synchronized in S-phase by release from DTB were immunoprecipitated with FLAG M2 beads. Immunoprecipitated samples were analyzed by western blotting with indicated antibodies. (B) LRR1ΔVHL mutant, unable to interact with CUL2, can still interact with the replisome. Chromatin extracts of FLAG-LRR1 or FLAG-LRR1ΔVHL-expressing cells synchronized in S-phase by release from DTB were immunoprecipitated with FLAG M2 beads. Immunoprecipitated samples were analyzed by western blotting with indicated antibodies. The western blot signal was quantified for all proteins in IP samples and FLAG-signal in IP and input (chromatin fraction) and represented as a fold change over the WT FLAG-LRR1 underneath the western blot. (C) GINS was immunoprecipitated from chromatin extracts of FLAG-LRR1 or FLAG-LRR1ΔVHL-expressing cells, synchronized in S-phase (DTB release). Immunoprecipitated samples were analyzed by western blotting with indicated antibodies. The western blot signal was quantified for FLAG-signal in IP and input (chromatin fraction) and represented as a fold change over the WT FLAG-LRR1 underneath the western blot. (D) Endogenous mAC-tagged LRR1 can interact with replisome components on chromatin in S-phase. Chromatin extracts of mAC-LRR1-expressing cells, synchronized in S-phase (with lovastatin release), were enriched on GFP-Trap magnetic agarose. Immunoprecipitated samples were analyzed by western blotting with indicated antibodies. (E) CUL2 and endogenous LRR1 can interact with GINS on S-phase chromatin. Chromatin extracts of mAC-LRR1 expressing cells, synchronized in S-phase as in (D), were co-immunoprecipitated with GINS antibody after treatment with p97i (5 μM). Immunoprecipitated samples were analyzed by western blotting with indicated antibodies.

Having established methods to observe replisome disassembly and ubiquitylation of MCM7 during this process, we next set out to analyze the mechanism of this process. First, we wanted to determine whether the CUL2^LRR1^ ubiquitin ligase interacts with the replisome during S phase. Such interactions would support the model that CUL2^LRR1^ drives the ubiquitylation and unloading of the replisome at termination, suggested by phenotypes of CRISPR-Cas9-guided knockout of LRR1 in MCF10A cells.^16^ To do this, we transiently expressed FLAG-tagged LRR1 in U2OS cells and confirmed that it can interact with endogenous CUL2 through a FLAG immunoprecipitation (IP) (Figure S3A). Cells overexpressing this FLAG-LRR1 did not exhibit cell proliferation defects (Figure S3B), nor any differences to cell-cycle progression (Figure S3C). Following this, we confirmed that we could observe co-immunoprecipitation of the replisome components MCM7, CDC45, and GINS, with FLAG-LRR1 from S phase chromatin (Figure 3A).

In order to investigate this further and determine whether LRR1 needs to form a complex with CUL2 for interactions with terminated CMG on chromatin, we expressed FLAG-LRR1 or a mutant lacking the VHL box (FLAG-LRR1ΔVHL),^23^ which cannot interact with CUL2 (Figure S3A) and whose expression also did not affect cell proliferation or cell-cycle progression (Figures S3B and S3C). FLAG-tagged recombinant LRR1 proteins (WT and ΔVHL) were immunoprecipitated from S phase chromatin, and the resulting material was analyzed for interactions with CMG components: MCM2, MCM7, and CDC45 (Figure 3B). FLAG-LRR1ΔVHL could interact with chromatin and the replisome, but its interaction with the replisomes appeared somewhat destabilized (Figure 3B). We also performed a reciprocal experiment through immunoprecipitation of GINS from the S phase chromatin fraction of cells expressing FLAG-LRR1 or FLAG-LRR1ΔVHL (Figure 3C). In this case again, we observed that FLAG-LRR1ΔVHL could interact with the CMG helicase, suggesting that formation of complex with CUL2 subunit is not essential for LRR1 substrate recognition. Interestingly, in both types of experiments, we can observe a higher level of chromatin-bound FLAG-LRR1ΔVHL in comparison with normal FLAG-LRR1 protein, suggesting that the kinetics of interaction is affected by the lack of enzymatic activity of CUL2^LRR1^ complex.

Unfortunately, endogenous LRR1 is expressed at a very low level in cells, and we struggled to detect it reproducibly in either whole-cell extracts or the chromatin fractions with a number of commercially available antibodies or antibodies raised by ourselves (data not shown). To detect endogenous LRR1 more efficiently, we decided therefore to use CRISPR-Cas9 to generate conditional auxin-inducible degron cells using HCT116 parental cell lines, tagging endogenous LRR1 N-terminally with the mAC tag (mini AID tag fused to mClover; mAC-LRR1) (Figure S4A). The auxin-inducible degron facilitates rapid protein degradation upon exposure to the plant hormone auxin, while mClover allows for enrichment of tagged LRR1 protein using the GFP-trap system. Firstly, we verified the bi-allelic gene tagging of LRR1 through genomic PCR (Figure S4B) and determined that tagging of LRR1 at the N terminus did not affect the proliferation of cells (Figure S4C). Of note, tagging LRR1 at the C terminus with a similar tag did not produce viable bi-allelically tagged clones, which suggested that the protein’s function is impaired (data not shown).

Using GFP-trap beads and enriching for mAC-LRR1, we could confirm that mAC-LRR1, expressed at endogenous levels, interacts with replisome components and CUL2 on chromatin during S phase (Figure 3D). We also performed the reciprocal experiment and found that immunoprecipitated GINS from S phase chromatin could interact with CUL2 and mAC-LRR1 (Figure 3E).

### Cullin ubiquitin ligase inhibitor (CULi) traps replisomes on chromatin in S phase and G2

Having shown that CUL2 and LRR1 interact with the replisome, we next wanted to determine whether the activity of this ubiquitin ligase is required for replisome disassembly in human somatic cells. Firstly, we used the small-molecule inhibitor, MLN4924 (hereafter referred to as CULi), which inhibits the neddylation pathway, primarily blocking the activation of all Cullin-type ubiquitin ligases.^30^ It acts rapidly and does not require much previous cell manipulation to downregulate protein levels and has been used in several previous studies with model systems, including *Xenopus* egg extracts and mouse embryonic stem cells.^4^^,^^6^^,^^9^^,^^10^^,^^14^

As DNA replication termination and replisome disassembly happen continuously throughout S phase, inhibition of this process should therefore lead to higher levels of replisome components on chromatin in S phase, and prolonged retention of the replisome on chromatin as cells enter into G2/M stage of the cell cycle. This may potentially perturb S phase progression if the recycling of replisome components is essential for completion of S phase.^16^ General inhibition of replication fork progression (replication stress) can also lead to similar phenotypes, as forks stall and fewer termination events occur, which makes it difficult to specifically distinguish between these two scenarios.

**Figure 4:**
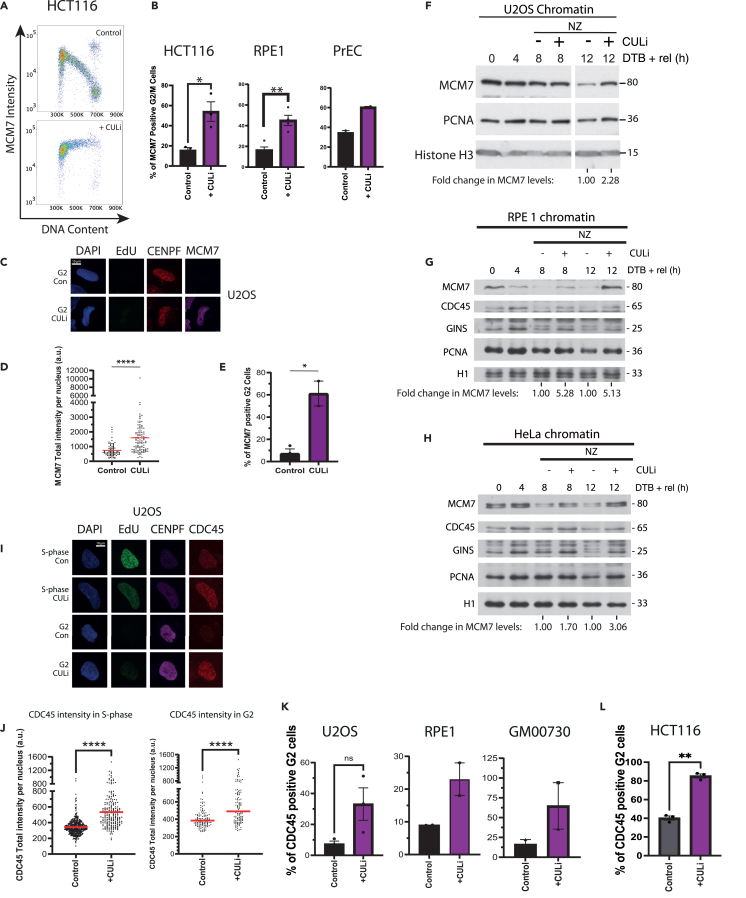
Cullin ligase inhibitor leads to replisome retention on the chromatin (A) MCM7 accumulates on chromatin upon CULi treatment. Asynchronous HCT116 cells were treated with CULi for 6 h. Cells were harvested and extracted with CSK buffer to visualize MCM7 bound to chromatin. Example FACS plots for the total MCM7 intensity (y axis) against DNA content (x axis) in HCT116 cells. Shown are untreated control (top) and +CULi (bottom). (B) Quantification of the percentage of G2/M cells positive for MCM7 in HCT116 cells from (A) (*n* = 3) (*p* = 0.0187, unpaired t test), but also from RPE1 cells (see Figure S5B) (*n* = 4) (*p* = 0.0026, unpaired t test) and PrEC cells (see Figure S5C) (*n* = 2). All mean ± SEM. (C) MCM7 accumulates on chromatin upon CULi treatment; representative immunofluorescence images of chromatin-bound EdU, CENPF, and MCM7 in G2-phase U2OS cells in asynchronous population ± CULi. (D) Quantification of (C): chromatin-bound MCM7 intensity in CENPF-positive/EdU-negative cells (AVG > 30 cells/sample). Red lines indicate the median (*n* = 3) (p=<0.0001, Two-tailed Mann Whitney test). (E) Quantification of (C): the total proportions of G2 cells positive for MCM7, mean ± SEM (*n* = 3) (*p* = 0.0121, Two-tailed unpaired t test). (F) MCM7 accumulates on chromatin upon CULi treatment in synchronized U2OS cells. U2OS cells were synchronized with DTB and released for indicated times ± CULi ± nocodazole (NZ). Cells were extracted with CSK buffer and chromatin samples analyzed through western blotting with indicated antibodies. (G) Same as for (F) but in RPE1 cells. (H) Same as for (F) but in HeLa cells. (I) CDC45 accumulates on chromatin upon CULi treatment - representative immunofluorescence images of chromatin-bound EdU, CENPF, and CDC45 in S-phase and G2-phase U2OS cells in asynchronous population ± CULi. (J) Quantification of (I): total chromatin-bound CDC45 intensity in EdU-positive (S-phase) (AVG > 100 cells/sample) and CENPF-positive (G2) cells (AVG > 35 cells/sample) (*n* = 3). Red lines indicate the median (p=<0.0001 for both, Two-tailed Mann Whitney test). (K) Quantification of (I): the total proportion of G2 U2OS cells positive for CDC45 (*n* = 3) (*p* = 0.0727, Two-tailed paired t test), mean ± SEM. Also for RPE1 cells (*n* = 2) (AVG > 40 cells/sample) and GM00730 cells (*n* = 2) (AVG > 35 cells/sample). (L) CDC45 accumulates on chromatin upon CULi treatment in HCT116 cells. Cells were synchronized in mid S-phase (24 h lovastatin and 14 h mevalonic acid release) and treated ± CULi for 2 h, followed by WEE1i for 1 h before cells were fixed and analyzed with QIBC (AVG > 4175 cells/sample). Shown is the quantification of the proportion of G2-phase HCT116 cells positive for CDC45, *n* = 3, mean ± SEM shown, (*p* = 0.0083, two-tailed Student’s t test).

To investigate the effects of Cullin inhibition, we treated asynchronous cell cultures with a range of 1–10 μM CULi for 6 h, allowing time for cells in S phase to progress into G2/M stage of the cell cycle so that we can observe any replisome retention. This treatment led to an accumulation of MCM7 on chromatin in cells with G2/M DNA content, seen through FACS analysis in HCT116 and U2OS cell lines, with 1 μM CULi having no effect in U2OS cells (Figure S5A). We then confirmed this retention of MCM7 on G2 chromatin using 5 μM CULi through FACS, immunofluorescence microscopy, and western blotting of extracted nuclei (Figures 4A–4H) across a number of different cancerous: HCT116 (colon), U2OS (bone), and HeLa (cervical), and non-cancerous: RPE1 (human hTERT immortalized retinal pigment epithelial cells) and PrEC (human prostate epithelial cells), cell lines.

In order to visualize the active CMG complexes more specifically and exclude dormant MCM2-7 complexes from our analysis, we next measured levels of chromatin-bound CDC45. Using western blotting and immunofluorescence microscopy, we could observe accumulation of this CMG component on chromatin in both S phase and G2-phase cells upon CULi treatment (Figures 4G–4L, S5B, and S5C), albeit to a lesser extent than MCM7. Again, we confirmed this observation in a number of cell lines: cancerous HeLa, U2OS, and HCT116 and immortalized RPE1 and GM00730 (human dermal fibroblasts) cells.

All the aforementioned results support the role of Cullin-type ubiquitin ligases in replisome disassembly in S phase. However, cell-cycle analyses of cells treated with CULi revealed an accumulation of cells with G1/early S phase DNA content (Figure S6A−S6C) as well as a reduction in the number of mitotic cells (Figure S6D), suggesting cell-cycle inhibition. CULi is inhibiting the activity of *all* Cullin-type ubiquitin ligases, and, specifically in the early stages of DNA replication, it is inhibiting CUL4-driven CDT1 degradation^31^ and CUL3-driven Cyclin E degradation.^32^ Stabilization of CDT1 is known to cause re-loading of MCM2-7 onto chromatin, re-replication, and checkpoint activation,^21^^,^^30^ while Cyclin E overexpression has been shown to cause replication stress through increased replication-transcription collisions.^33^ Indeed, we do see stabilization of both CDT1 and Cyclin E in our cells (Figure S6E), as well as very high levels of chromatin-bound MCM7 visible in our FACS plots upon CULi treatment across both S phase and G2/M cells (Figures 4A, S5B, and S5C).

Consistent with replication problems and G1/S arrest, we observe reduced EdU incorporation into nascent DNA (Figure S6F–S6G) and delayed S phase progression, as cells treated with CULi struggle to complete DNA synthesis within the same time frame as control cells (8 h) (Figure S6H). Cells treated with CULi also exhibit elevated levels of DNA synthesis (EdU incorporation) in a subset of G2 cells (Figure S6I), suggesting that either DNA synthesis was not finished in S phase or some G2 cells can undergo re-replication.^34^^,^^35^ Finally, similarly to previous reports,^21^^,^^36^ we can detect an induction of S phase checkpoint upon CULi treatment in the form of checkpoint kinase CHK1 phosphorylation (S345) (Figure S6J), while concomitant inhibition of the S phase checkpoint master-kinase ATR (+ATRi) is able to partially rescue the delay in S phase progression (Figure S6H). Altogether, our data demonstrate that CULi has multiple effects: it leads to additional loading of MCM2-7 onto chromatin while impeding the unloading of active CMG helicases, cumulatively inducing DNA replication stress and S phase checkpoint activation. Given these side effects, more specific methods are therefore required for assessing the function of CUL2^LRR1^ in replisome disassembly.

### CUL2 depletion leads to retention of replisomes on chromatin in S phase and G2

**Figure 5:**
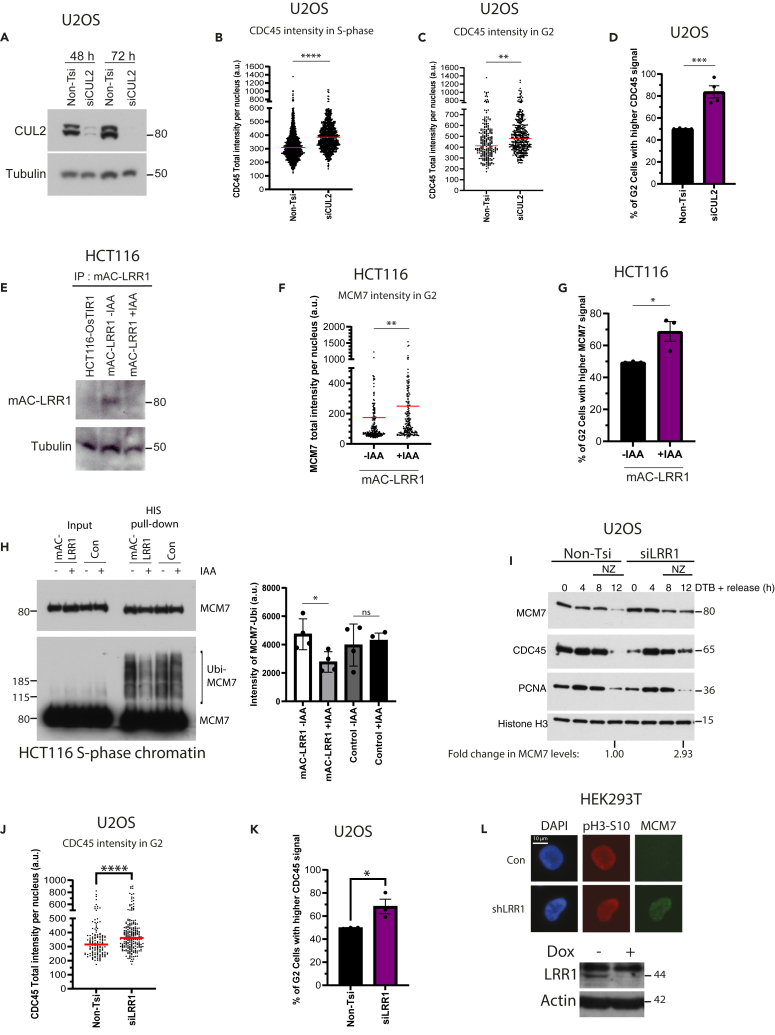
CUL2 or LRR1 depletion leads to replisome retention on the chromatin (A) CUL2 downregulation by siRNA. U2OS cells were transfected with Non-T or CUL2 siRNA (two sequences used in combination; see Oligonucleotides in key resources table) for 48 or 72 h. Whole-cell extract samples were then analyzed by western blotting with the indicated antibodies. (B) CDC45 accumulates on chromatin in S-phase upon depletion of CUL2. Asynchronous U2OS cells transfected with Non-T or CUL2 siRNA for 72 h were analyzed by immunofluorescence. Shown is the quantification of total chromatin-bound CDC45 intensity in EdU-positive (S-phase) cells (*n* = 4) (AVG > 295 cells/sample). Red lines indicate the median (p=<0.0001, Two-tailed Mann-Whitney test). (C) Same as for (B) but in CENPF-positive (G2) cells (*n* = 4) (*p* = 0.0076, Two-tailed Mann-Whitney test) (AVG > 80 cells/sample). (D) Same as for (C) but quantification of the percentage of G2 cells with CDC45 signal above the median of Non-Tsi level (*n* = 4) (*p* = 0.0009, Two-tailed unpaired t test); mean value +/− SEM. (E) Degradation of mAC-LRR1 after 24 h of auxin treatment. Whole-cell extracts of HCT116-OsTIR1 cells expressing mAC-LRR1 were incubated with GFP-Trap Magnetic Agarose and samples analyzed by western blotting with indicated antibodies. (F) MCM7 accumulates on chromatin in G2 cells upon mAC-LRR1 depletion. HCT116 LRR1-mAC cells were treated with DOX for 48 h and ±IAA for 24 h and analyzed by immunofluorescence. Shown is the quantification of chromatin-bound MCM7 intensity in CENPF-positive (G2) cells (*n* = 3) (AVG > 70 cells/sample). Red lines indicate the median (p=<0.0001, Two-tailed Mann-Whitney test). (G) Same as for (F) but quantification of the percentage of G2 cells with MCM7 signal above the median of -IAA cells (*n* = 3) (*p* = 0.0359, Two-tailed unpaired t test); mean value +/− SEM. (H) Ubiquitylation of MCM7 on chromatin is reduced in S-phase following LRR downregulation. HCT116 LRR1-mAC cells were treated with DOX for 48 h, transfected with HIS-Ubi plasmid, synchronized with STB and released for 6 h ± IAA. HIS-tagged proteins were isolated using the HIS pull-down assay and samples analyzed by western blotting with the indicated antibodies. Levels of MCM7 ubiquitylation were then quantified using ImageJ. Mean ± SEM (*n* = 4) (*p* = 0.0145, Two-tailed paired t test). (I) CMG components accumulate on chromatin in synchronized cells upon LRR downregulation. U2OS cells were transfected with Non-T or LRR1 siRNA (see Oligonucleotides in key resources table), synchronized with DTB and then released for indicated time points, with addition of NZ at 8 h. Chromatin was extracted with CSK buffer and samples analyzed by western blotting with indicated antibodies. (J) CDC45 accumulates on chromatin in G2 cells upon LRR1 downregulation. Asynchronous U2OS cells transfected with Non-T or LRR1 siRNA for 72 h were analyzed by immunofluorescence. Shown is the quantification of total chromatin-bound CDC45 intensity in CENPF-positive (G2) cells (*n* = 3) (p=<0.0001, Two-tailed Mann-Whitney test) (AVG > 60 cells/sample). Red lines indicate the median. (K) Same as for (J) but quantification of the percentage of G2 cells with CDC45 signal above the median of Non-Tsi control cells (*n* = 3) (*p* = 0.0417, Two-tailed unpaired t test); mean value +/− SEM. (L) MCM7 accumulates on G2 chromatin upon LRR1 downregulation. Whole-cell extracts of HEK293T cells inducibly expressing LRR1 shRNA (see STAR Methods) for 6 days were analyzed by western blotting with indicated antibodies. Also shown is the representative immunofluorescence images of chromatin-bound pH3-S10 and MCM7 in G2-phase cells expressing LRR1 shRNA.

To target the CUL2^LRR1^ ligase more specifically, we next decided to deplete CUL2 in U2OS cells with small interfering RNA (siRNA) (Figure 5A). Long-term CUL2 downregulation (3–5 days) caused a reduced rate of cell proliferation (Figure S7A) and a slight accumulation of cells in S phase with FACS analysis, suggesting slowed cell-cycle progression (Figure S7B). Furthermore, we could also detect a reduction of EdU incorporation into nascent DNA through immunofluorescence (Figure S7C), suggesting replication stress akin to that observed with CULi—a phenotype not previously reported for CUL2 depletion. Despite the lower rate of DNA synthesis, the immunofluorescence analysis of chromatin-bound CDC45 levels in these cells revealed a significant increase in both S phase (EdU-positive) and G2 (EdU-negative/CENPF-positive) cells (Figure 5B–5D), suggesting that the role of CUL2 in replisome disassembly is conserved in human cells. The S phase defects we observe could thus be a consequence of replisome disassembly defects, i.e., an inability to recycle components, but, to investigate this further, we decided to more specifically target the substrate receptor LRR1.

### LRR1 is important for replisome disassembly during S phase

In order to downregulate LRR1, we made use of our HCT116 LRR1 degron cells (mAC-LRR1). Short-term knockout of LRR1 using CRISPR-Cas9 was shown previously to lead to the accumulation of CDC45 on chromatin in S phase.^16^ To validate this in our hands, we first showed that adding indole-3-acetic acid (auxin, IAA) to the growth media indeed caused LRR1 degradation. Following enrichment of mAC-LRR1 on GFP-trap beads, after 24 h exposure of cells to 500 μM auxin, we could observe downregulation of mAC-LRR1 (Figure 5E). Akin to CUL2 depletion, cells deleted of mAC-LRR1 displayed a ∼50% reduction in viability following long-term deletion (7–10 days) (Figure S4D), although we did not observe an altered cell-cycle profile with a short-term depletion (Figure S4E). Upon analyzing the effect of mAC-LRR1 degradation on replisome unloading in S phase, we also found an increased level of MCM7 on chromatin in cells in G2 phase of the cell cycle (Figures 5F and 5G). To ascertain that the retainment of MCM7 on chromatin is the result of reduced ubiquitylation of MCM7, we expressed HIS-tagged ubiquitin in control or in mAC-LRR1 cells and undertook HIS pull-down from chromatin of S phase-arrested cells. We could indeed detect a strong reduction in MCM7 ubiquitylation upon degradation of LRR1 (Figure 5H).

Finally, to confirm these results in other cell lines, we downregulated LRR1 expression using siRNA or small hairpin RNA (shRNA) approaches. Firstly, U2OS cells depleted of LRR1 with siRNA were synchronized in early S phase and released. Using this approach, we found that LRR1 depletion causes a prolonged association of CMG components on chromatin through western blotting (Figure 5I), in the absence of visible changes in S phase progression (Figure S7D). In non-synchronized cells we could also detect an increase in CDC45 on chromatin in G2 cells using immunofluorescence (CENPF-positive) (Figures 5J and 5K), with no reduction in EdU incorporation (Figure S7E). Next, we generated HEK293T cells, which express an inducible pool of shRNA against LRR1, causing an 80%–90% reduction in the level of LRR1 mRNA by 6 days using quantitative PCR (Figure S7F). In HEK293T cells we can also sometimes detect endogenous LRR1, due to higher levels of the protein, and could therefore also detect a reduction in the steady-state levels of the protein (Figure 5L). Importantly, depletion of LRR1 with shRNA in this way also led to retainment of MCM7 on chromatin in cells with a low level of histone H3-S10 phosphorylation, representing late G2 cells (Figure 5L). Altogether, cells with depleted LRR1 have shown fewer problems with cell-cycle progression and progression through S phase, most likely reflecting the smaller pool of substrates affected by downregulation of LRR1, compared with CUL2. The lack of signs of replication stress suggests that the observed retention of CMG components on chromatin in S and G2 stages of the cell cycle more likely indicates direct problems with replisome disassembly, rather than global replication defects.

### p97 inhibition (p97i) traps replisomes on chromatin but leads to many cellular problems

**Figure 6:**
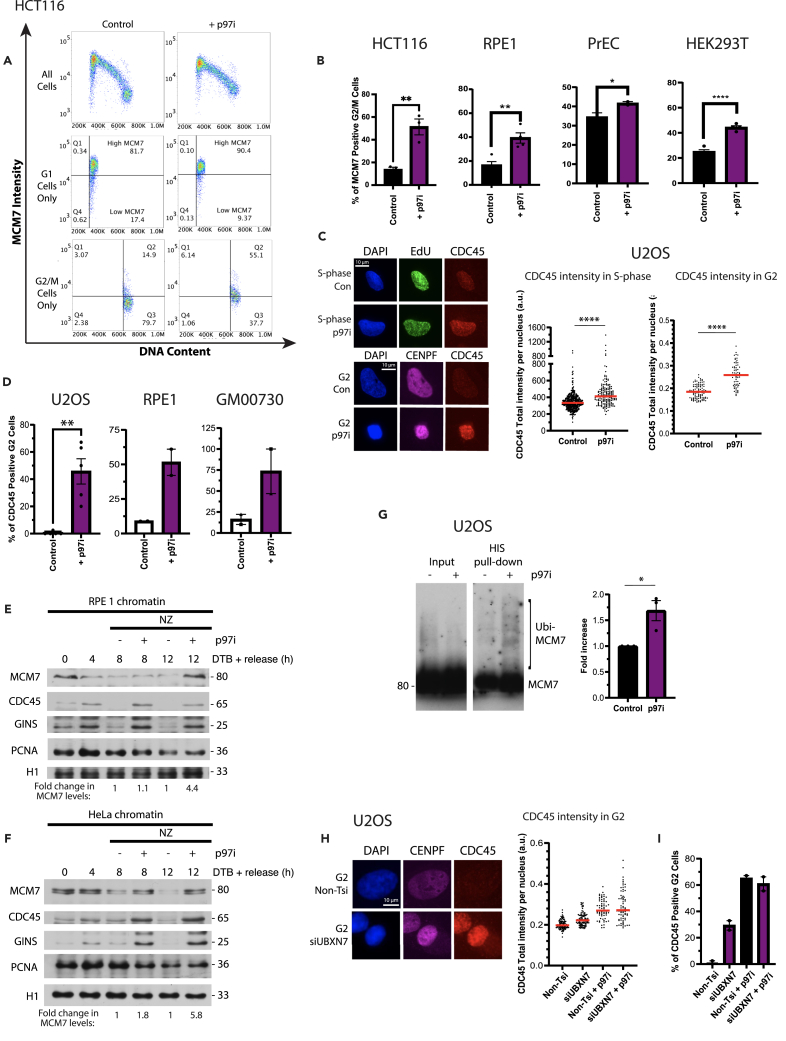
p97 inhibition leads to replisome retention on the chromatin (A) p97i treatment affects loading and unloading of MCM7 from chromatin. Flow cytometric analysis of the MCM7 chromatin binding pattern following 6 h p97i treatment in asynchronous HCT116 cell culture. Shown are representative plots showing total MCM7 intensity (y axis) against DNA content (x axis). Overall binding pattern throughout the cell cycle (top), gated examples of G1 cells only (middle) and gated examples of G2/M cells only (bottom), used for quantification. (B) Quantification of (A): percentage of G2/M cells positive for MCM7 in HCT116 (*n* = 3) (*p* = 0.0068, unpaired t test). Also for RPE1 (see Figure S8A) (*n* = 4) (*p* = 0.0051, unpaired t test), PrEC (see Figure S8A) (*n* = 2) (*p* = 0.0491) and HEK293T cells (see Figure S8A) (*n* = 4) (p=<0.0001, unpaired t test). All mean ± SEM. (C) CDC45 accumulates on chromatin in S-phase and G2 phase upon p97i treatment - representative immunofluorescence images of chromatin-bound EdU and CDC45 in EdU-positive (S-phase) and chromatin-bound CENPF and CDC45 in CENPF-positive (G2) U2OS cells from asynchronous population, treated ± p97i for 6 h. Also shown is quantification of chromatin-bound CDC45 intensity in EdU-positive (S-phase) (*n* = 3) (AVG > 96 cells/sample) and CENPF-positive (G2) (*n* = 3; relative to nuclei size) (AVG > 35 cells/sample) cells. Red lines indicate the median (p=<0.0001 for both, Two-tailed Mann-Whitney test). (D) Same as for (C) but quantification of the percentage of G2 cells positive for CDC45 in U2OS treated with p97i (*n* = 3) (*p* = 0.0074, Two-tailed paired t test) (AVG > 35 cells/sample), RPE1 (*n* = 2) (AVG > 35 cells/sample) and GM00730 cells (*n* = 2) (AVG > 45 cells/sample); mean value +/− SEM. (E) Replisome components accumulate on chromatin upon p97i treatment in synchronized RPE1 cells. RPE1 cells were synchronized with DTB and released for indicated time points ± p97i ± nocodazole (NZ). Cells were extracted with CSK buffer and chromatin samples analyzed through western blotting with indicated antibodies. (F) Same as for (E) but in HeLa cells. (G) Ubiquitylation of MCM7 on chromatin in S-phase is increased following p97i. U2OS cells were transfected with HIS-Ubi plasmid, synchronized with STB and released for 6 h ± p97i. HIS-tagged proteins were isolated using the HIS pull-down assay and samples analyzed by western blotting with the indicated antibodies. Levels of MCM7 ubiquitylation were quantified using ImageJ; mean ± SEM (*n* = 3) (*p* = 0.0243, Two-tailed unpaired t test). (H) CDC45 accumulates on chromatin in G2 upon UBXN7 depletion; representative immunofluorescence images of chromatin-bound CENPF and CDC45 in G2-phase U2OS cells from asynchronous population, treated ± UBXN7 siRNA (see Oligonucleotides in key resources table) for 72 h. Also shown is quantification of chromatin-bound CDC45 intensity in CENPF-positive (G2) cells, relative to nuclei size (*n* = 2) (AVG > 75 cells/sample). Red lines indicate the median. (I) Same as for (H) but quantification of the total proportion of G2 cells positive for CDC45 (*n* = 2); mean value +/− SEM.

Next, we went on to investigate the role of p97 in replisome disassembly, with the expectation that it works downstream of MCM7 ubiquitylation. To do this, we used the CB5083 inhibitor (p97i), as p97 inhibition has been shown previously to cause retention of the replisome on chromatin in model systems.^9^^,^^10^^,^^14^ In an analogous way to our studies with CULi, asynchronous cells were treated for 6 h with p97i to allow for cells to progress from S phase into G2/M. FACS analysis of these cells revealed that the 6 h treatment with p97i led to a slightly higher level of MCM7 signal on chromatin in cells with G2/M DNA content, but the general pattern of MCM7 unloading was not much changed (Figure 6A). This suggested that the unloading of dormant origins, comprising the majority of MCM2-7 present on chromatin, is not affected by p97 inhibition. We could observe an analogous result using a number of other cell lines: RPE1, PrEC, and HEK293T (FigureS 6B and S8A). We also observed a significant increase in CDC45 on chromatin in S phase and in G2-phase cells by immunofluorescence (Figure 6C). Again, the increased level of CDC45 in G2 phase of the cell cycle upon p97i treatment was confirmed across U2OS, RPE1, and GM00730 cell lines (Figure 6D). Finally, we can also see accumulation of MCM7, CDC45, and GINS on isolated chromatin in late S phase and G2 upon p97i treatment in RPE1 and HeLa cell lines by western blotting (Figures 6E and 6F). Altogether, these data show that while unloading of inactive MCM2-7 double hexamers is not affected by p97i, the unloading of CMGs is. Next, we performed an HIS-Ubiquitin pull-down assay with cells synchronized in early S phase and released for 6 h in the presence of p97i. With this, we can see an increase of ubiquitylated MCM7 on chromatin, as expected from previous reports in model systems^4^^,^^9^^,^^14^ (Figure 5G), although not as pronounced as described in embryonic model systems.

Intriguingly, the FACS profiles revealed a change in MCM7 chromatin binding in G2/M cells but also in G1 (Figure 6A). In the presence of p97i, in G1, we could detect only cells with fully licensed replication origins, and we appeared to lose the population of cells undergoing origin licensing (Figures 6A and S8B). The most likely cause of this loss of MCM2-7 loading was perturbations to cell-cycle progression with cells struggling to progress from mitosis back into G1, resulting in overall fewer numbers of cells loading MCM2-7 hexamers. Importantly, this cell-cycle problem cannot be detected by analyzing the total DNA content of cells treated with p97i for 6 h (Figure S8C), so we decided to further investigate the effects of p97i on cell-cycle progression using immunofluorescence and FACS analysis. Firstly, we found a significant reduction in EdU incorporation into nascent DNA after 6 h of p97i treatment (Figure S8D),^16^ and cells treated with p97i were not able to complete DNA synthesis within an 8 h time frame unlike control cells, with EdU-pulsed cells struggling to progress beyond early-mid S phase (Figure S8E). Secondly, we observed a significant decrease in the total intensity of the G2 marker CENPF in all cells as well as visibly smaller G2 nuclei (Figures S8F and 6C), suggesting problems in G2 stage of the cell cycle. Finally, analysis of HCT116 cells positive for histone H3 S10 phosphorylation revealed clear reductions in mitotic cells upon treatment with p97i (Figure S8G), as previously observed.^16^ These data support the idea that p97i treatment causes defects during DNA synthesis and replication stress, resulting in fewer cells entering G2 and mitosis. p97 is proposed also to impair ATR signaling during S phase^37^; we observe therefore a very mild increase of CHK1 phosphorylation level upon p97i treatment (Figure S8H), while simultaneously observing an appearance of cells with higher levels of γH2AX staining (Figure S8I).^38^ Overall, our data suggest that p97i affects cell-cycle progression not only through S phase but also in every other stage of the cell cycle. As a result, we cannot detect changes in the overall cell-cycle distribution when treating an asynchronous culture with p97 inhibitor.

Because of the pleiotropic effects of p97 inhibition, we next went on to determine whether we could more specifically impede the function of p97 in replisome disassembly by depleting the p97 cofactor, UBXN7, as we and others have shown that UBXN7 facilitates replisome disassembly by p97 during S phase in *Xenopus* egg extract.^12^ Depletion of UBXN7 with siRNA for 72 h did indeed cause an accumulation of CDC45 on chromatin in G2 cells (Figure 6H), but without causing defects in EdU incorporation (Figure S8J). Notably, CDC45 accumulation in this case was to a lesser extent than with p97i, which is to be expected as UBXN7 is known to be a facilitator of p97 function, rather than being essential, and downregulation of UBXN7 was causing less replication stress.^12^ Reassuringly, levels of CDC45 accumulation are not additive when UBXN7 siRNA and p97i are combined (Figure 6I), which supports a model in which they work together to promote replisome disassembly, as suggested previously.^15^

Altogether, in relation to the function of p97 in replisome disassembly, we can detect higher levels of CDC45 and MCM7 on chromatin in cells with S- and G2/M DNA content and we see an increase of ubiquitylated MCM7 on chromatin in S phase. It is important however to keep in mind that these phenotypes are likely a result of several problems during DNA replication, and not only due to a defect in replisome disassembly. By depleting the p97 cofactor UBXN7 however, we were able to avoid the pleiotropic effects of p97 inhibition and observe a more specific defect in CMG disassembly, suggesting that UBXN7 facilitates p97 in this process.

### Replisome disassembly in mitosis

**Figure 7:**
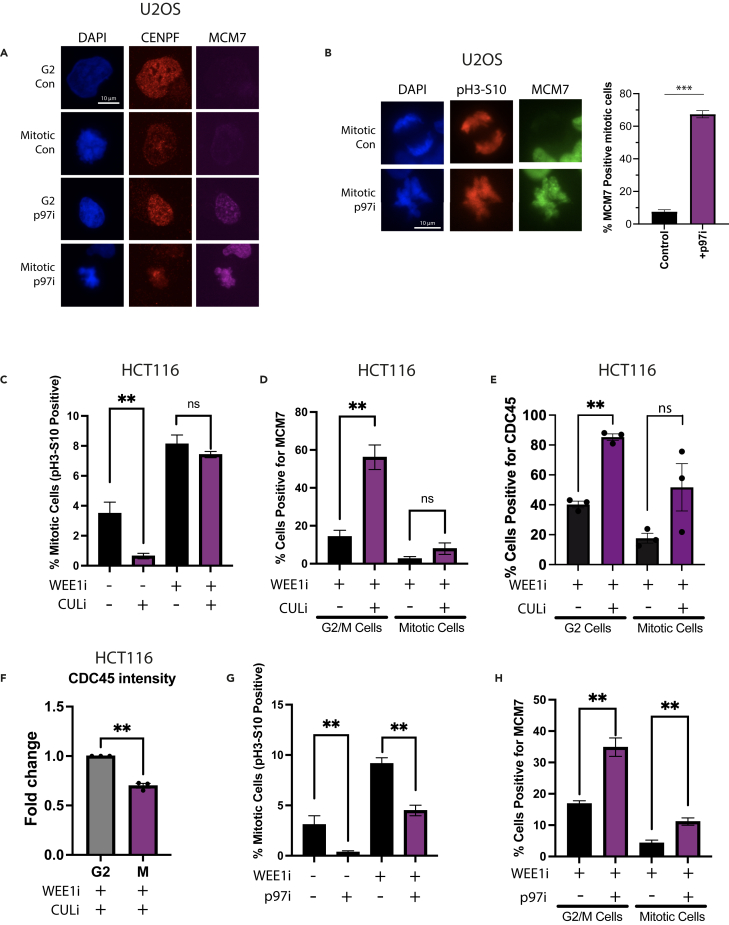
The mitotic replisome disassembly pathway is active in human somatic cells (A) MCM7 is present on condensed mitotic chromosomes following p97i treatment. Representative immunofluorescence images of chromatin-bound CENPF and MCM7 in G2 and mitotic U2OS cells from asynchronous population, treated ± p97i for 6 h. (B) MCM7 is present on condensed mitotic chromosomes following p97i treatment - representative immunofluorescence images of chromatin-bound pH3-S10 and MCM7 in mitotic U2OS cells from asynchronous population, treated ± p97i for 6 h with quantification of the percentage of pH3-S10-positive cells, which are positive for MCM7 (*n* = 3) (*p* = 0.0003, Two-tailed paired t test); mean value +/− SEM. (C) Effects of CULi in combination with WEE1i on cell cycle progression. Asynchronous HCT116 cells were treated for 6 h ± CULi and supplemented for the final hour ± WEE1i. Histogram displays quantification of the percentage of mitotic cells (pH3-S10-positive) (*n* = 3) (*p* = 0.0015, unpaired t test); mean value +/− SEM. (D) MCM7 accumulated on chromatin following CULi treatment is unloaded from chromatin in mitosis. Analysis of the percentage of cells retaining MCM7 in G2/M and mitosis following 6 h CULi treatment and 1 h WEE1i treatment, with FACS analysis. Shown are quantification of the cells, with G2/M DNA content, positive for MCM7 staining on chromatin (*p* = 0.0045, unpaired t test) and mitotic cells (pH3-S10 positive) positive for MCM7 staining on chromatin (ns) (*n* = 3 for all); mean value +/− SEM. (E) CDC45 accumulated on chromatin following CULi treatment is unloaded from chromatin in mitosis. Analysis of the percentage of cells retaining CDC45 in G2/M and mitosis. HCT116 cells were synchronized in G1 (24 h lovastatin) and released for 14 h with mevalonic acid. At this point, cells were treated with ±CULi for 2 h, followed by 1 h WEE1i treatment. Shown are quantification of the cells, with G2/M DNA content (AVG > 4175 cells/sample), positive for CDC45 staining on chromatin (*p* = 0.0083) and mitotic cells (AVG > 2100 cells/sample) positive for CDC45 staining on chromatin (ns, two-tailed Student’s t test) (*n* = 3 for all); mean value +/− SEM. (F) Same as for (E) but quantification of fold change in levels of average CDC45 intensity in G2 and mitotic cells (*n* = 3) (*p* = 0.002, two-tailed Student’s t test); mean value +/− SEM. (G) Effects of p97i in combination with WEE1i on cell cycle progression. Cells treated as in (C) but with p97i instead of CULi. Histogram displays quantification of the proportion of mitotic cells (pH3-S10-positive) (*n* = 3 for all) (-WEE1: *p* = 0.00709; +WEE1i: *p* = 0.00137, unpaired t test); mean value +/− SEM. (H) When p97i-treated cells are pushed into mitosis they still retain MCM7 on chromatin. Analysis of the percentage of cells retaining MCM7 in G2/M and mitosis following 6 h p97i treatment and 1 h of WEE1i treatment. Shown are quantification of the cells with G2/M DNA content positive for MCM7 (-WEE1i: *p* = 0.0043, unpaired t test) and mitotic cells (pH3-S10 positive) positive for MCM7 (+WEE1i: *p* = 0.0099) (*n* = 3 for all); mean value +/− SEM.

Finally, given our previous studies using the *Xenopus* model system,^11^ we decided to investigate the existence of a mitotic replisome disassembly pathway in human somatic cells. As shown earlier, treating cells with CULi or p97i impairs cell-cycle progression through S phase and leads to reduced proportions of cells, which are pH3-S10 positive. Despite small numbers of cells, we could see indications of MCM7 on chromatin in mitotic cells treated with p97i using immunofluorescence. These cells were identified by presenting condensed chromatin and kinetochore staining by CENPF or a strong signal for pH3-S10 (Figures 7A and 7B).

To be able to visualize the mitotic pathway more quantitively, we decided to push cells treated with p97i or CULi past the G2/M checkpoint arrest and into mitosis, through inhibition of the WEE1 kinase, which normally works to prevent mitotic entry by deactivating CDK1.^39^ First, we confirmed that a short treatment with WEE1 inhibitor (MK-1775, WEE1i) can push cells into mitosis without affecting the cell cycle or the MCM7 unloading pattern (Figure S9A). We next added WEE1i for the last hour of 6 h p97i/CULi treatments. Firstly, inhibition of WEE1 in +CULi cells pushed them through into mitosis as efficiently as control cells (Figure 7C). Analysis of MCM7 unloading from chromatin confirmed our previous data as there were significantly more cells with G2/M DNA content retaining MCM7 on chromatin when cells were treated with both CULi and WEE1i, but now we could also specifically analyze mitotic cells. Interestingly, although we could still detect some increase of MCM7 on chromatin in pH3-S10-positive cells, it was minor and not statistically significant (Figure 7D). Cells treated with CULi accumulate a lot of MCM2-7 on chromatin due to origin re-licensing and inhibition of replisome unloading. They do however lose these complexes from their chromatin when they enter mitosis, indicating a very efficient mitotic unloading pathway. To analyze directly the unloading of specifically the CMG complexes, we decided to follow the fate of CDC45. CDC45 immunostaining is much weaker than MCM7. To enrich our samples in cells at the right stage of the cell cycle, we arrested cells in G1 with lovastatin and released them into S phase. Once the majority of cells entered S phase, we treated them with CULi for 2 h following with 1 h of WEEi inhibitor treatment. The level of chromatin-bound CDC45 and pH3-S10 was quantified. The CULi treatment increased the number of cells retaining CDC45 on chromatin in G2 stage of the cell cycle (4N DNA content, no pH3-S10), but we could observe much lower numbers of cells retaining CDC45 on chromatin in mitosis (pH3-S10 positive) in both control and CULi-treated cells (Figures 7E and 7F).

On the other hand, inhibition of WEE1 in p97i-treated cells was much less effective, pushing ∼50% fewer cells into mitosis than the control (Figure 7G). Analysis of MCM7 unloading from chromatin confirmed our previous data as there were significantly more cells with G2/M DNA content retaining MCM7 on chromatin when cells were treated with both p97i and WEE1i, but now, unlike with CULi, we could also detect a significant increase in the number of mitotic (pH3-S10 positive) cells retaining MCM7 (Figure 7H). This observation suggests that the mitotic replisome unloading pathway requires p97 segregase activity. Additionally, the previously described loss of G1 MCM7 loading was also rescued through WEE1i treatment, indicating that, despite the persisting reduction to the proportions of mitotic populations, some cells were now able to cycle through to the G1 stage of the cell cycle (Figure S9B). Altogether, these experiments suggest that the mitotic replisome disassembly pathway is indeed conserved in human cells and that p97 activity is important for unloading of the replisomes in mitosis.

## Discussion

As a process indispensable for the propagation of life, the mechanisms involved during all stages of DNA replication are well conserved throughout eukaryotic evolution. In agreement with this, we present data to suggest that the core mechanisms of replisome disassembly, first reported by our lab and others using model organisms,^3^^,^^4^^,^^6^^,^^9^^,^^10^^,^^11^^,^^13^^,^^14^^,^^18^ are similarly active in human somatic cells. However, the specific characteristics of cell cycle and S phase regulation of somatic cells mean that explicit analysis of replication termination and replisome disassembly is particularly challenging. As a result, several of the tools commonly utilized for the study of DNA replication termination in model systems are less suitable for use in somatic cell models.

We first confirmed that we can observe the S phase-specific ubiquitylation of MCM7 on chromatin, including with K48-linked ubiquitin chains, analogous to that reported in model systems.^3^^,^^4^ The role of CUL2^LRR1^ in replisome disassembly is well described in model systems, and our data established that their mechanism of action was likely conserved into human somatic cells. Immunoprecipitation of either LRR1 or components of the active replisome confirmed both CUL2^LRR1^ complex formation and the ability to interact with the replisome. Interestingly, we have shown that mutant LRR1 that cannot interact with CUL2 can still interact with the terminated replisome, suggesting that LRR1 does not require prior formation of the CUL2^LRR1^ complex to bind to its substrate. This is consistent with the cryoelectron microscopy (Cryo-EM) structure suggesting that LRR1 is the main part of CUL2^LRR1^ interacting with the replisome.^5^

Following this, we compared different methods of inhibition of CUL2^LRR1^ activity in various cell lines and established that both CUL2 and LRR1 are essential for replisome disassembly in S phase and MCM7 ubiquitylation. Analogously, short-term CRISPR-guided knockout of LRR1 in both MCF10A and RPE1 cells was also shown to induce CDC45 accumulation on chromatin in late S phase and reduce cell proliferation through defects to late S phase progression,^16^ while a combination of CULi inhibitor treatment and LRR1 knockdown was required to achieve this in mouse embryonic stem cells.^10^ Altogether, these data indicate that CUL2^LRR1^ is indeed the ubiquitin ligase driving replisome disassembly in human somatic cell lines, and the reduction of proliferation and S phase progression problems likely represent the consequence of the loss of replisome unloading in otherwise unperturbed cell systems.

Our investigations also clearly show that small-molecule inhibitors affecting neddylation and activity of Cullins (CULi) cannot be used in human somatic cells to specifically target replisome disassembly, although successfully used for this purpose both in model organisms and in mouse ES cells.^4^^,^^6^^,^^9^^,^^10^^,^^14^ Even short treatments of human cell lines with such inhibitors lead to replication stress and inhibition of S phase progression, which agree with previous reports of the loss of Cullin4-mediated protein degradation.^21^^,^^30^

We have also investigated the involvement of p97 segregase in the process of replisome disassembly using the small molecule p97 ATPase inhibitor. Again, as with CULi, our results indicate increased retention of MCM7 and CDC45 on chromatin in S and G2 phases of the cell cycle but with an accompanying increase in levels of ubiquitylated MCM7 on chromatin. However, we do also observe induction of replication stress upon this treatment. p97 is thought to have a plethora of substrates and has been implicated in numerous mechanisms, including the DNA damage response, DNA replication, autophagy, and apoptosis.^38^^,^^40^^,^^41^^,^^42^ Our data indicate that a short-term p97i treatment leads to the complete loss of cell-cycle progression, not initially detected through the analysis of total DNA content, as every stage of the cell cycle is affected. Importantly, cells treated with p97i cannot progress through S phase in a timely manner. p97 with its cofactor FAF1 has been shown previously to be important for prevention of replication stress through regulation of CDT1, origin re-licensing, and firing.^43^ This could possibly explain some of our observed phenotypes, even though the level of MCM2-7 present on chromatin upon p97 inhibition does not match the levels observed upon Cullin inhibition, which leads to stimulation of origin re-licensing. Another possible path of p97 activity during S phase could be regulation of MRE11 activity at reversed forks, as p97 was shown to prevent MRE11 retention and excessive DNA resection at double-strand breaks sites.^44^ Finally, it has also been suggested that the loss of p97 results in vast increases to the numbers of ubiquitylated protein complexes on chromatin potentially forming barriers to replisome progression.^43^^,^^45^ Altogether, p97 has a number of roles, which, when inhibited, could lead to the observed replication stress. To investigate the specific function of p97 in replisome disassembly, a more process-specific tool is required. We have shown therefore that the UBXN7 cofactor of p97, which facilitates replisome disassembly in *Xenopus laevis* egg extract,^12^^,^^15^ is also important for replisome unloading from chromatin in human cells, while not leading to strong impediment in S phase progression.

Despite the explained aforementioned effects of small-molecule inhibition to cell-cycle progression, we were able to identify small numbers of mitotic cells upon p97 inhibition that retained MCM7 on chromatin. Furthermore, this population of cells was enhanced by inhibiting WEE1 kinase, which negatively regulates mitotic entry through CDK1 phosphorylation.^46^ This allowed us to gain an insight into the secondary mitotic, backup pathway for replisome disassembly. During mitosis, any replisomes retained on chromatin from S phase (either terminated or stalled) are thought to be ubiquitylated by the E3 ubiquitin ligase TRAIP, resulting in their removal from chromatin by p97.^6^^,^^8^^,^^11^ We found that treatment with CULi and WEE1i efficiently releases cells from G2 arrest and allows them to progress into mitosis. CULi leads to a large accumulation of MCM2-7 complexes on chromatin in S phase due to re-licensing and inhibition of replisome disassembly. Nevertheless, strikingly, most of the MCM7 retained on chromatin from S phase appeared to be disassembled in mitosis. This suggested that the backup pathway of disassembly is functional and very efficient in somatic cells. Conversely, when we combined p97i and WEE1i, only a proportion of cells managed to progress into mitosis. In these mitotic cells, replisomes were retained across both S phase and mitosis, with a similar fold of enrichment upon p97i treatment. This is indicative of the important role played by the p97 segregase in both pathways.

In conclusion, our work establishes that the two mechanisms of replisome disassembly uncovered in model organisms are indeed conserved in human somatic cell lines. We have generated tools and assays allowing us to study these processes but also show that care needs to be taken when interpreting data as it is very challenging to specifically target these processes without other interferences.

### Limitations of the study

As discussed throughout this manuscript, the phenotypes observed as a result of inhibition of replisome disassembly are very similar to ones expected to be observed as a result of induction of replication stress. Much care and many additional controls are needed to ascertain that observed accumulation of replisomes on chromatin is indeed representing retained terminated replisomes rather than stalled replisomes unable to converge. Downregulations of LRR1 and UBXN7 are the most specific tools we found thus far to directly affect the process of replisome disassembly.

Although we have used both male- and female-derived cell lines, the influence of the sex of the cell lines on the results of the study cannot be reported and so is a limitation to the research’s generalizability.

## STAR★Methods

### Key resources table

**Table:** 

REAGENT or RESOURCE	SOURCE	IDENTIFIER
**Antibodies**		

Rabbit polyclonal anti-CENPF	Abcam	Cat#ab5; RRID: AB_304721
Mouse monoclonal anti-MCM7 (clone 141.2)	Santa Cruz	Cat#Sc-9966; RRID: AB_627235
Rabbit polyclonal anti-MCM7 (clone H-300)	Santa Cruz	Cat#Sc-22782; RRID: AB_2142817
Mouse monoclonal anti-Mitosin (clone 11)	Fisher Scientific	Cat#15805639
Rabbit monoclonal anti-CDC45 (clone D7G6)	Cell Signaling Technology	Cat#1181S
Mouse monoclonal anti-CDC45 (clone G-12)	Santa Cruz	Cat#Sc-55569; RRID: AB_831146
Rat anti-CDC45	Heinz-Peter Nasheuer	N/A
Rabbit monoclonal anti-Phospho-Histone H3 (Ser10) (clone D2C8)	Cell Signaling Technology	Cat#3377S
Mouse monoclonal anti-Phospho-Histone H2AX (Ser139) (clone JBW301)	Merck Millipore	Cat#05-636; RRID: AB_309864
Mouse monoclonal anti-BrdU (clone B44)	BD Biosciences	Cat#347580; RRID: AB_10015219
Mouse monoclonal anti-FLAG (clone M2)	Sigma-Aldrich	Cat#F3165; RRID: AB_259529
Mouse monoclonal anti-Cyclin E (clone E−4)	Santa Cruz	Cat#Sc-377100; RRID: AB_2923122
Mouse monoclonal anti-PCNA	Sigma-Aldrich	Cat#P8825; RRID: AB_477413
Rabbit polyclonal anti-Histone H3	Cell Signaling Technology	Cat#9715S
Rabbit monoclonal anti-K48-linkage specific Polyubiquitin (clone D9D5)	Cell Signaling Technology	Cat#12805S
Sheep anti-GINS	In house	N/A
Rabbit monoclonal anti-CUL2 (clone EPR3104(2))	Abcam	Cat#166917
Mouse monoclonal anti-MCM2 (clone 46/BM28)	BD Biosciences	Cat#610700; RRID: AB_2141952
Mouse monoclonal anti-Tubulin (clone DM1A)	Sigma-Aldrich	Cat#T9026
Rat monoclonal anti-GFP (clone 3H9)	ChromoTek	Cat#3H9; RRID: AB_10773374
Rabbit polyclonal anti-LRR1	Atlas	Cat#HPA069364; RRID: AB_2686129
Rabbit polyclonal anti-CDT1 (clone H-300)	Santa Cruz	Cat#Sc-28262; RRID: AB_2076885
Mouse monoclonal anti-Beta Actin (clone C4)	Santa Cruz	Cat#Sc-47778; RRID: AB_626632
Rabbit polyclonal anti-Phospho CHK1 (Ser345)	Cell Signaling Technology	Cat#2341S
Goat polyclonal anti-mouse IgG, Alexa Fluor 488	Invitrogen	Cat#A-32723; RRID: AB_2633275
Goat polyclonal anti-mouse IgG, Alexa Fluor 647	Invitrogen	Cat#A-21235; RRID: AB_2535804
Goat polyclonal anti-rabbit IgG, Alexa Fluor 555	Invitrogen	Cat#A-21428; RRID: AB_2535849
Goat polyclonal anti-mouse IgG, HRP	Sigma-Aldrich	Cat#A5278; RRID: AB_258232
Goat polyclonal anti-rabbit IgG, HRP	Sigma-Aldrich	Cat#A9161
Donkey polyclonal anti-sheep IgG, HRP	Sigma-Aldrich	Cat#A3415; RRID: AB_258076
Rabbit polyclonal anti-rat IgG, HRP	Sigma-Aldrich	Cat#A9542; RRID: AB_258456

**Chemicals, peptides, and recombinant proteins**		

MLN4924	Stratech Scientific	Cat# S7109-SEL
CB5083	Generon Ltd	Cat# B6032
AZD6738	Generon Ltd	Cat# B6007
Doxycycline hyclate	VWR International Ltd	Cat# CAYM14422
Tetracycline hydrate	Sigma-Aldrich	Cat# T3258
3-Indole Acetic Acid	Sigma-Aldrich	Cat# I2886
MK-1775	Cayman Chemicals	Cat#21266
Puromycin	Thermo Fisher Scientific	Cat# A1113803
Hygromycin B GOLD	Thermo Fisher Scientific	Cat#10687010
Aphidicolin	VWR International	Cat#178273-1
Nocodazole	Thermo Fisher Scientific	Cat#15997255
Fugene HD transfection reagent	Promega UK Ltd	Cat# E2312
Hoescht 33258	Sigma-Aldrich	Cat#94403
Propidium Iodide	Scientific Laboratory Supplies	Cat# P4864-10ML
5-Bromo-2′-deoxyuridine (BrdU)	Sigma-Aldrich	Cat#B5002
Fluoroshield with DAPI	Sigma-Aldrich	Cat#F6057
Thymidine	Sigma-Aldrich	Cat#T9250
Lovastatin	Acros Organics	Cat#AO398529
Mevalonic Acid	Sigma-Aldrich	Cat#BCBX4250
2′Chloroacetamide	Scientific Laboratory Supplies	Cat#C0267
Thiazolyl Blue Tetrazolium Bromide	Thermo Fisher Scientific	Cat# L11939.06
Dharmafect 1 transfection reagent	Horizon Discovery	Cat#T-2001-03
TransIT®-2020 transfection reagent	Mirus Bio	Cat#MIR 5404

**Critical commercial assays**		

Click-iT™ EdU Cell Proliferation Kit for Imaging, Alexa Fluor™ 488 dye	Thermo Fisher Scientific	Cat#C10337
Dynabeads™ His-Tag Isolation and Pulldown	Thermo Fisher Scientific	Cat#10103D
ANTI-FLAG® M2 Affinity Gel	Merck Millipore	Cat#A2220
Dynabeads™ Protein A for Immunoprecipitation	Invitrogen	Cat#10001D
GFP-Trap® Magnetic Agarose	ChromoTek	Cat#gtma

**Experimental models: Cell lines**		

Human: U2OS cells	ATCC	ATCC: HTB-96, RRID: CVCL_0042
Human: HCT116 cells	ATCC	ATCC: CCL-247, RRID: CVCL_0291
Human: GM00730	Coriell Institute for Medical Research	Fibroblast from skin: GM00730, RRID: CVCL_L944
Human: HeLa	ATCC	ATCC: CRM-CCL-2, RRID: CVCL_0030
Human: HEK293T	ATCC	ATCC: CRL-3216, RRID: CVCL_0063
Human: hTERT RPE1	ATCC	ATCC: CRL-4000, RRID: CVCL_4388
Human: immortalised prostate primary epithelium cells (PrEC)	Robert G. Bristow	N/A

**Oligonucleotides**		

siRNA targeting sequence: human CUL2 (individual)	Horizon Discovery	Cat#D-007277-01-0005
siRNA targeting sequence: human CUL2 (individual)	Horizon Discovery	Cat# D-007277-03-0005
siRNA targeting sequence: human LRR1 (smartpool)	Horizon Discovery	Cat#L-016820-01-0005
siRNA targeting sequence: human UBXN7 (smartpool)	Horizon Discovery	Cat#L-023533-02-0005
Primer: LRR1 qPCR Forward: GGGACCCGCTATGAGCTAAG (5′- 3′)	This paper	N/A
Primer: LRR1 qPCR Reverse: CCTTTAACCGAACAGTGGCTTT (5′- 3′)	This paper	N/A
Primer: mAID (confirming mAC-LRR1) Forward: ATCTTTAGGACAAGCACTCTTCTCC (5′-3′)	This paper	N/A
Primer: LRR1 (confirming mAC-LRR1) Forward: AGGAATTGCCAAGTCAAATACAG (5′-3′)	This paper	N/A
Primer: LRR1 (confirming mAC-LRR1) Reverse: AGGGAGAATATTGTGGGAGAAAG (5′-3′)	This paper	N/A
Guide RNA: LRR1 N-terminus: TGTAGCTTCATCTCGCCCAA (5′-3′)	This paper	N/A
TRIPZ Lentiviral Inducible LRR1 shRNA - 1 ATGAGAGCCATATGGAATC (5′- 3′)	Horizon Discovery	Clone Id: V2THS_25090
TRIPZ Lentiviral Inducible LRR1 shRNA - 2 CAAACACAAATTTTTGCGG (5′- 3′)	Horizon Discovery	Clone Id: V3THS_339461
TRIPZ Lentiviral Inducible LRR1 shRNA - 3 CTCCCTTAGCTCATAGCGG (5′- 3′)	Horizon Discovery	Clone Id: V3THS_339460
TRIPZ Lentiviral Inducible LRR1 shRNA – 4 TCTCCCTTAGCTCATAGCG (5′- 3′)	Horizon Discovery	Clone Id: V3THS_339458
TRIPZ Lentiviral Inducible LRR1 shRNA – 5 TTTGCGGTATCCAAATCTT (5′- 3′)	Horizon Discovery	Clone Id: V3THS_339456
TRIPZ Lentiviral Inducible LRR1 shRNA - 6 TAGACAGATATCCACAGGA (5′- 3′)	Horizon Discovery	Clone Id: V3THS_339457

**Recombinant DNA**		

Plasmid: pMD2.G	Addgene	Cat#12259
Plasmid: psPAX2	Addgene	Cat#12260
Plasmid: POG44	Thermo Fisher Scientific	Cat#V600520
Plasmid: MCM7 pCDNA5-FRT-TO-FA-3xflagMcm7	Diffley’s Lab, Francis Crick Instutute	N/A
Plasmid: MCM4 pCDNA5-FRT-TO-FA-3xflagMcm4	Diffley’s Lab, Francis Crick Instutute	N/A
Plasmid: FLAG-hLRR1	Starostina et al.^23^	N/A
Plasmid: FLAG-hLRR1ΔVHL	Starostina et al.^23^	N/A
Plasmid: pX330-U6-Chimeric_BB-CBh-hSpCas9	Addgene	Cat#42230
Plasmid: PX330 CRISPR plasmid containing gRNA targeting N-terminus of LRR1	This paper	Addgene Deposit 84419
Plasmid: pBluescript II	Stratagene	Cat#212205
Plasmid: pBluescript containing LRR1 homology sequence	This paper	N/A
Plasmid: pMK345: mAID-mClover tag with Hygromycin resistance marker	Addgene	Cat#121179
Plasmid: DONOR plasmid for making mAC-LRR1 degrons, LRR1 homology arms in pMK345	This paper	Addgene Deposit 84419
Plasmid: pcDNA5 His-ub-wt	Ron Hay’s lab, Dundee	N/A
Plasmid: pcDNA5 His-ub-K48R	This paper	Addgene Deposit 84419

**Software and algorithms**		

ImageJ	Schneider et al.^29^	https://imagej.nih.gov/ij/
FlowJo™ (v10.7.1)	BD Life Sciences	https://www.flowjo.com/
CellProfiler (v4.0.6)	Kamentsky et al.^47^ and Carpenter et al.^48^	https://cellprofiler.org/releases
GraphPad Prism (v9.2.0)	GraphPad	https://www.graphpad.com/scientific-software/prism/www.graphpad.com/scientific-software/prism/
HCS Studio Cell Analysis	Thermo Fisher Scientific	https://www.thermofisher.com/uk/en/home/life-science/cell-analysis/cellular-imaging/high-content-screening/hcs-studio-2.html
Spotfire (v10.5.0.72)	Tibco	https://www.spotfire.com/
RStudio Team (2020) (v4.2.2)	RStudio: Integrated Development for R. RStudio, PBC, Boston, MA URL	http://www.rstudio.com/

### Resource availability

#### Lead contact

Further information and requests for resources and reagents should be directed to and will be fulfilled by the lead contact, Aga Gambus (a.gambus@bham.ac.uk).

#### Materials availability

Plasmids generated in this study have been deposited to Addgene: DONOR plasmid for making mAC-LRR1 degrons; PX330 CRISPR plasmid containing gRNA targeting N-terminus of LRR1; and pcDNA5 His-ub-K48R.

#### Data and code availability

Data: Microscopy data reported in this paper will be shared by the lead contact upon request.

Code: This paper does not report original code.

Additional information: Any additional information required to reanalyse the data reported in this paper is available from the lead contact upon request.

### Experimental model and study participant details

#### U2OS

U2OS (RRID: CVCL_0042) is a cell line with epithelial morphology that was derived in 1964 from a moderately differentiated sarcoma of the tibia of a 15-year-old, White, female osteosarcoma patient (ATCC). This cell line was grown in DMEM +10% FBS (Sigma F7524) (Tet-free for DOX-inducible lines (PAN Biotech UK Ltd P30-3602)) + 1% pen/strep (Thermo Fisher Scientific 15140122) + 2 mM L-Glutamine (Thermo Fisher Scientific 25030081) at 37°C with 5% CO_2_. Cell line not authenticated.

U2OS FLAG-MCM7 and FLAG-MCM4 cells were generated using the U2OS Flp-In cell line, as previously described.^49^

#### HCT116

The HCT116 cell line (RRID: CVCL_0291) has epithelial morphology and was isolated from the colon of an adult male with colon cancer (ATCC). This cell line was grown in McCoys 5A media +10% FBS (Tet-free for DOX-inducible lines) + 1% pen/strep +2 mM L-Glutamine at 37°C with 5% CO_2_. Cell line not authenticated.

HCT116 Tet-OsTIR1 cells were grown as detailed above.

HCT116 Tet-OsTIR1 LRR1-mAC degron cells were generated as detailed in^50^ and grown in conditions detailed above. Bi-allelic gene tagging was confirmed using genomic PCR, see.^50^ Genomic PCR was carried out using GoTaq HotStart Green MasterMix (Promega), as per manufacturer’s instruction, using the following primers: mAID Forward Primer ATCTTTAGGACAAGCACTCTTCTCC (5′-3′), LRR1 Forward: AGGAATTGCCAAGTCAAATACAG (5′-3′), LRR1 Reverse: AGGGAGAATATTGTGGGAGAAAG (5′-3′).

#### GM00730

The GM00730 fibroblast cell line (RRID: CVCL_L944) was isolated from skin of the arm of an apparently healthy 45-year-old female (Coriell Institute for Medical Research; a kind gift from Dr Grant Stewart). This cell line was grown in DMEM +20% FBS +1% pen/strep +2 mM L-Glutamine at 37°C with 5% CO_2_. Cell line not authenticated.

#### HeLa

HeLa (RRID: CVCL_0030) is a cell line with epithelial morphology that was isolated from the cervix of a 31-year-old, Black female with adenocarcinoma (ATCC). This cell line was grown in DMEM +10% FBS +1% pen/strep +2 mM L-Glutamine at 37°C with 5% CO_2_. Cell line not authenticated.

#### HEK293T

HEK293T (RRID: CVCL_0063) is a cell line exhibiting epithelial morphology. This cell line is a highly transfectable derivative of the HEK293 cell line that was isolated from the kidney of a female human embryo, into which the temperature-sensitive gene for SV40 T-antigen was inserted (ATCC). This cell line was grown in DMEM +10% FBS +1% pen/strep +2 mM L-Glutamine at 37°C with 5% CO_2_. Cell line not authenticated.

HEK293T cells expressing tet-inducible LRR1 shRNA were generated by lentiviral infection, as detailed in Chiang et al.^51^ and grown in conditions described above. Briefly, for lentiviral production, low passage HEK293T cells were transfected with pTRIPZ-shRNALrr1 (a set of six sequences, Horizon Discovery, detailed in Table S1) and appropriate packaging vectors (pMD2.G and psPAX2), using TransIT-2020 Transfection Reagent (Mirus Bio 5404), following manufacturer’s instructions. Cells were cultured for 24 h before harvesting viral supernatant. U2OS cells were then transduced by centrifuging with viral supernatant containing 8 μg/mL polybrene (Sigma) for 90 min at 2500 rpm. Cells were finally selected with 1 μg/mL Puromycin.

#### hTERT RPE1 (RPE1)

The hTERT RPE1 (referred to as RPE1) cell line (RRID: CVCL_4388) has epithelial-like morphology and is an hTERT-immortalised derivative of a normal 1-year old female human retinal pigment epithelial (RPE-340) cell line^52^ (ATCC). These cells were grown in DMEM-F12 + 10% FBS +1% pen/strep +2 mM L-Glutamine at 37°C with 5% CO_2_. Cell line not authenticated.

#### Immortalized human prostate primary epithelium cells (PrEC)

The immortalized male human prostate primary epithelium cells (PrEC)^53^ (a kind gift from Prof. R.G Bristow) were cultured in Keratinocyte Serum Free Media (Gibco 17005-034) supplemented with 2.5 μg of Epidermal Growth Factor (Gibco 10450-013), 25 μg of bovine pituitary extract (Gibco 13028-013) + 1% pen/strep at 37°C with 5% CO_2_. To passage, PrEC cells were washed briefly in PBS and incubated with Trypsin-EDTA for 5–10 min. Cells were then harvested in KSFM containing approximately 20 μg/mL Trypsin inhibitor (Roche 10109886001). Cells were subsequently centrifuged and re-suspended in fresh KSFM to remove residual trypsin and trypsin inhibitor. Cell line not authenticated.

### Method details

#### Cell synchronisation

Double thymidine block (DTB) – exponentially growing cells were treated with 2.5 mM thymidine (Merck Life Science Ltd 6060) for 16 h to arrest them at G1/early S phase, washed 3× with PBS, grown in fresh media for 8 h, treated a second time with 2.5 mM thymidine for 16 h, washed 3× with PBS and released into fresh media for indicated time points. Single thymidine block (STB) – exponentially growing cells were treated with 2.5 mM thymidine for 16 h to arrest them at G1/early S phase, washed with PBS 3× and released into fresh media for indicated time points. Nocodazole (NZ) – exponentially growing cells were treated with 3.3 μM nocodazole (Fisher Scientific Ltd 15997255) for 24 h to arrest them at Mitosis. Lovastatin - exponentially growing cells were treated with 20 μM Lovastatin (Acros Organics AO398529) for 24 h to arrest them at G1 phase. This is followed by 12 h treatment with 2 mM Mevalonic Acid (Sigma BCBX4250) for synchronous release into S phase.

#### Plasmids

For generation of mAC-LRR1-degron cells, CRISPR AID plasmids were generated as detailed in.^54^ CRISPR-Cas9 was expressed from the pX330-U6-Chimeric_BB-CBh-hSpCas9 plasmid^55^ (Addgene 42230) and targeted the N-terminus (first Methionine) of LRR1 using the complimentary guide RNA TGTAGCTTCATCTCGCCCAA (5′-3′). Donor plasmids were also generated as detailed in.^54^ Briefly, we cloned homology arms upstream and downstream of the CRISPR target region into the pBluescript backbone (∼500 bp each). After inverse PCR, the homology arms of LRR1 were cloned into a cassette containing the mAC tag and hygromycin resistance marker pMK345 for N-terminal tagging^56^ was cloned to generate the donor plasmid. For the HIS pull-down assay, we transfected cells with pcDNA5-6HIS-Ubiquitin-wildtype or pcDNA5-6HIS-Ubiquitin-K48R. For generation of FLAG-MCM7 and FLAG-MCM4 Flp-In cell lines, we used pcDNA5-FRT-TO-1xFLAG-MCM7, pcDNA5-FRT-TO-1xFLAG-MCM4 plasmids and the POG44 plasmid. cDNAs encoding human LRR1 were obtained from pCDNA3-FLAG-LRR1. The pCDNA3-FLAG-LRR1Δ construct was described previously.^23^

#### Plasmid transfection for transient expression

Cells were seeded at experiment-dependent concentrations and 24 h later they were transfected with an empirically determined optimal concentration of plasmid DNA, using Fugene HD transfection reagent (Promega UK Ltd E2312). The manufacturer’s protocol was followed and a Fugene HD transfection reagent:DNA ratio of 1:3 was used.

#### Flow cytometry

Following described inhibitor treatments, cells were fixed with 70% ethanol in PBS for 16 h at −20°C (typically unextracted cells for BrdU and EdU detection), or with 4% paraformaldehyde in PBS for 15 min at RT (following CSK extractions). For antibody staining, cells were washed twice in Washing buffer (5% BSA, 0.1% Tween 20, PBS), then re-suspended in 100 μL primary antibody in Washing buffer and incubated at RT for 1 h, with rocking to prevent cells from settling. The cells were then washed twice in Washing buffer and re-suspended in 100 μL secondary antibody in Washing buffer for 1 h at RT in the dark. Stained cells were washed 1× in Washing buffer and 2× in PBS before being re-suspended in either Hoescht Staining Buffer (5 μg/mL Hoescht 33258, PBS) or Propidium Iodide Staining Buffer 25 μg/mL Propidium Iodide, 50 μg/mL RNase A, PBS).

For BrdU detection, 10 μM of BrdU was added to the growth media 1 h prior to harvesting. Cells were collected and fixed in ethanol as described. Fixed cells were washed once in PBS before being resuspended in 1 mL 2 M HCL supplemented with 0.1 mg/mL Pepsin for 20 min. Cells were then washed, and antibody staining carried out as described.

For EdU detection, 10 μM of EdU was added to the growth media for the indicated lengths of time prior to harvesting. Cells were collected and fixed in ethanol as described. Fixed cells were washed once in PBST and cells re-suspended in 100 μL EdU Click-It reaction (Thermo Fisher Scientific C10337) for 30 min. Cells were washed again in PBST and cell pellets re-suspended in 500 μL Propidium Iodide Staining Buffer, as above.

To explore the replisome binding pattern on chromatin, cells were extracted using CSK buffer (10 mM HEPES, pH7.4, 300 mM sucrose, 100 mM NaCl, 3 mM MgCl2, 0.5% Triton X-100, 1 μg/mL of each aprotinin, leupeptin and pepstatin, 1 mM PMSF) to remove soluble fractions. The protocol used to extract cells has been described elsewhere.^57^ Cells were subsequently analyzed with a Beckman Cytoflex instrument. Details for antibodies are described within the key resources table and dilutions used are in Table S2.

#### Immunofluorescence microscopy

Cells were seeded directly onto pre-sterilised glass cover slips, placed into individual wells of a 6-well plate at experiment-dependent concentrations. 24 h later they were treated with inhibitors or 50 nM siRNA. Cells were pulse-labelled with 10 μM EdU for 20 min, before being extracted with CSK buffer (see ‘flow cytometry’ section) for 10 min, to remove soluble fractions, and fixed with 4% PFA for 10 min. EdU staining was performed using the Click-It EdU kit (Thermo Fisher Scientific C10337) and manufacturer’s instructions, before cells were washed twice with Blocking buffer (1% BSA, 0.1% Tween 20) and incubated with the primary antibody for 1 h in the dark. After two washes with PBS and two washes with the Blocking buffer, cells were incubated with the secondary antibody for 2 h in the dark. Coverslips were then mounted onto slides using Fluoroshield + DAPI mounting medium, to co-stain for DNA. Samples were imaged using a Leica DM6000 upright widefield microscope using the 40×/1.25 Plan Apo objective. Details for antibodies are described within the key resources table and dilutions used are in Table S2.

#### Quantitative image-based microscopy (QIBC)

Samples were prepared as described in the ‘immunofluorescence microscopy’ section but images were acquired on a CellInsight CX5 High Content Screening Platform (Fisher Scientific) with 10X objective. A minimum of 5,000 cells for each experimental condition were captured. Details for antibodies are described within the key resources table and dilutions used are in Table S2.

#### Chromatin isolation

Harvested cells were washed in PBS twice before extraction in 300 μL cold CSK buffer (see ‘flow cytometry’ section) for 15 min. Samples were centrifuged at 5000 rpm for 5 min, before the soluble nucleoplasm fraction was collected. Pellets were then re-suspended in another 1 mL cold CSK buffer and centrifuged again at 7000 rpm for 7 min. The final chromatin pellets were solubilised in UTB buffer (8 M Urea, 50 mM Tris-HCl pH 7.5, 150 mM β-Mercaptoethanol) and sonicated (25% amplitude – 2 × 10 s using a SONICS Vibra Cell VCX 130PB ultrasonic liquid processor). Chromatin lysates were clarified by centrifugation at max speed for 10 min and the protein concentration determined by Bradford method. Finally, they were mixed with 1× NuPAGE LDS sample buffer (Fisher Scientific NP0008), boiled for 5 min and analyzed by western blotting as described.

#### Whole cell lysate preparation

To validate LRR1 degradation in mAC-LRR1 expressing cells by western blotting, cells were lysed in UTB buffer (8 M Urea, 50 mM Tris-HCl pH 7.5, 150 mM β-Mercaptoethanol) and western blotting was performed as described. To confirm CUL2 depletion with siRNA, 7 × 10^5^ cells were pelleted and resuspended in 300 μL 1× RIPA buffer (50 mM Tris pH8, 150 mM NaCl, 1% Triton X-100, 0.5% sodium deoxylcholate, 0.1% SDS). Samples were vortexed every 10 min for 30 min, then sonicated (25% amplitude – 2 × 10 s using a SONICS Vibra Cell VCX 130PB ultrasonic liquid processor) and centrifuged (max speed, 10 min, 4°C). Clarified lysates were mixed with 1× NuPAGE LDS sample buffer, boiled for 5 min and analyzed by western blotting as described.

#### HIS-ubiquitin pull down

For each sample, U2OS cells were seeded into 2 × 150mm dishes at 1.5 × 10^5^ cells/ml (30 × 10^5^ cells total). 24 h later they were transfected with 0.5 μg/mL pcDNA5-HIS-Ubi plasmid using Fugene HD. 32 h later, they were treated with 2.5 mM thymidine for 16 h. After 2× PBS washes, cells were treated with DMSO or p97i for 6 h, before 3 × 10^6^ cells were harvested for each sample (determined with a Countess II (Thermo Fisher Scientific) cell counter). Cells were extracted with CSK buffer (see ‘flow cytometry’ section) + 20 mM chloroacetamide for 15 min on ice before pellets were re-suspended in 300 μL US buffer (8 M urea, 50 mM sodium phosphate, pH8) and samples boiled at 95°C for 5 min. Following sonication (25% amplitude – 3 × 15 s using a SONICS Vibra Cell VCX 130PB ultrasonic liquid processor) and centrifugation (max speed, 10 min, 4°C), 250 μL of the clarified lysate was mixed with 150 μL HIS-Tag isolation dynabeads (Invitrogen 10103D) and 750 μL US buffer for 2 h, with rotation at RT. Beads were finally washed 5× with Wash buffer at RT (8 M urea, 50 mM Tris, pH6.8, 100 mM NaCl, 20 mM imidazole, 0.02% Tween 20, adjusted to pH6) and boiled in 2× NuPAGE LDS sample buffer before the original lysates (input) and the eluates (HIS pull-down) were analyzed by western blotting as described.

#### FLAG, GINS and GFP-TRAP immunoprecipitations

Immunoprecipitation of chromatin bound mAC-LRR1 and GINS from mAC-LRR1 expressing cells was carried out as follows. Cells were seeded at a density of 10^7^ cells/150mm dish. For efficient detection of replication termination complexes on the chromatin during S phase by western blotting their accumulation was enhanced by supplementing the cell culture media with CB5083 (5 μM) and AZD6738 (5 μM) for 4 h before harvesting cells. Harvested cells were washed in PBS twice before extraction in cold CSK buffer (see ‘flow cytometry’ section) + 20 mM chloroacetamide for 15 min. Lysates were clarified by centrifugation at 1500 × g for 5 min. Chromatin pellets were solubilised in cold chromatin lysis buffer (1% NP40, 10 mM Tris pH 7.5, 100 mM KOAc, 10% glycerol, 1mM MgCl_2_, Universal Pierce Nuclease) followed by sonication at high amplitude for 2.5 min (30s ON 30s OFF) using a Diagenode Bioruptor Standard Sonicator. Chromatin lysates were clarified by centrifugation at max speed for 5 min followed by protein quantification by Bradford method. Where appropriate, chromatin lysates were pre-cleared by incubation with isotype control IgG coated Dynabeads at 4°C with rotation for 1 h. For pulling down FLAG-tagged protein, 1 mg chromatin lysates were incubated with 50 μL FLAG M2 beads (Merck A2220) for 2 h at 4°C with rotation. For pulling down GINS, 1 mg chromatin lysates were incubated with 50 μL Dynabeads Protein A (Invitrogen 10001D) coated with 10 μg GINS antibodies for 2 h at 4°C with rotation. For pulling down mAC-LRR1, 1 mg chromatin lysates were incubated with 25 μL GFP-Trap Magnetic Agarose (Chromotek) for 2 h at 4°C with rotation. This was followed by washing beads thrice with washing buffer (50mM Tris-HCl pH 7.5, 100 mM KOAc, 1 mM MgCl_2_, 10% Glycerol, 0.1% NP40). The immunoprecipitated protein complexes were eluted by boiling beads in 2× NuPAGE LDS sample Buffer at 70°C for 10 min with shaking.

#### Western blotting

Proteins were resolved on a 4–12% SDS-PAGE gel and transferred onto PVDF membranes (Millipore) using Criterion Blotter (Bio-Rad). The membranes were then blocked using 5% milk in TBST (50 mM Tris-HCl pH 8.0, 150 mM NaCl, 0.1% Tween 20) for 1 h and incubated with appropriate primary antibodies overnight at 4°C. The membranes were washed 2 × 10 min with TBST and incubated with respective HRP-conjugated secondary antibodies for 2 h at RT. After 2 × 10 min TBST washes, membranes were developed with WesternBright ECL Spray (Advansta). Details for antibodies are described within the key resources table and dilutions used are in Table S2.

#### Cell viability assays

Cells were seeded and treated with experiment-dependent conditions. To measure growth rate of cells by Trypan blue exclusion method, single cell suspensions were mixed with an equal volume of Trypan blue, and cells were counted using the automated cell counter Countess II (Thermo Fisher Scientific).

To measure growth rate by MTT assay, cells were seeded in a 96 well plate at a density of 200 cells/well and treated with experiment-dependent conditions. To measure growth rate at every 24 h, cell culture medium was replaced by medium containing Thiazolyl Blue Tetrazolium Bromide (0.5 mg/mL) (Thermo Fisher Scientific L11939.06) and incubated for 4 h at 37°C, 5% CO_2_. Insoluble formazan salts formed by viable cell populations were dissolved by incubation in DMSO for 10 min followed by measurement of absorbance at 570 nm.

For colony assays, asynchronous cells were diluted to a seeding density of 1 × 10^4^ cells/mL. Cells were seeded into individual wells of 6-well plates at the following concentrations: 250, 500, 750, 1000 total cells per well. Approximately 24 h later, cells were treated with 100 ng/mL doxycycline as appropriate, and optionally treated with 100 μM IAA 24 h later. Cells were incubated for 7–10 days, or until sufficient colonies were observed, replacing the media every 3–4 days. At this point, the cell media was removed, and cells stained with methylene blue (2% methylene blue in 50% ethanol) for 5 min at RT and the plates dried at RT overnight.

#### siRNA transfection

Cells were seeded at 5 × 10^4^ cells/mL and 24 h later transfected with 50 nM Non-targeting (Non-T, Horizon Discovery D-001810-10-05), CUL2 (Horizon Discovery D-007277-01-0005 and D-007277-03-0005), LRR1 (Horizon Discovery L-016820-01-0005) or UBXN7 (Horizon Discovery L-023533-02-0005) siRNA using Dharmafect 1 (Horizon Discovery T-2001-02), following manufacturer’s protocol. Effective depletion was determined by western blotting of whole cell extracts using appropriate antibodies.

#### qPCR

qPCR was performed using SYBR Green PCR Master Mix (Thermo Fisher Scientific 4309155), following manufacturer’s instructions, using LRR1 primers forward: GGGACCCGCTATGAGCTAAG (5′- 3′) and reverse: CCTTTAACCGAACAGTGGCTTT (5′- 3′).

#### Inhibitors and reagents

5 μM CULi (MLN4924, Stratech Scientific S7109-SEL. Dissolved in DMSO to generate 5 mM stock); 5 μM p97i (CB5083, Generon Ltd B6032. Dissolved in DMSO to generate 5 mM stock); 5–10 μM ATRi (AZD6738, Generon Ltd B6007. Dissolved in DMSO to generate 10 mM stock); 2 μg/mL DOX (doxycycline hyclate, VWR International Ltd CAYM14422. Dissolved in DMSO to generate 2 mg/mL stock); 1 μg/mL Tetracycline (tetracycline hydrate, Simga T3258. Dissolved in DMSO to generate 2 mg/mL stock); 100 μM IAA (3-Indole Acetic Acid, Sigma I2886. Dissolved in DMSO to generate a 500 mM stock); 5 μM WEE1i (MK-1775, Cayman Chemicals, 21266. Dissolved in DMSO to generate a 10 mM stock); 1 μg/mL Puromycin (Thermo Fisher Scientific A1113803. Dissolved in HEPES buffer; 10 mg/mL stock); 100 μg/mL Hygromycin B GOLD (Thermo Fisher Scientific 10687010. Dissolved in H_2_O; 50 mg/mL stock); 10 μM Aphidicolin (APH, VWR International 178273-1. Dissolved in DMSO to generate 10 mM stock).

### Quantification and statistical analysis

For all experiments, the number (n) of biological repeats, errors bars and statistical tests used are described in the figure legend. For statistical significance: ∗*p* < 0.05, ∗∗*p* < 0.01, ∗∗∗*p* < 0.001, ∗∗∗∗*p* < 0.0001.

For flow cytometry analysis: data analysis was performed with FlowJo (v10.7.1) and gating was performed on cell cycle profiles using the Create Gates on Peaks function. For all pairwise comparisons, unpaired t-tests were performed to determine statistical significance.

For immunofluorescence microscopy analysis: all images were exported as raw greyscale TIFs and analyzed using CellProfiler (v4.0.6).^47^^,^^48^ The primary objects of interest (nuclei) were initially identified and masked onto the appropriate antibody staining channel. Any changes to the respective signal of individual factors within the nuclei was then determined by measuring the integrated intensity (a.u). XY dot plots of replication factors (EdU, MCM7 or CDC45) vs. CENPF were generated using Prism (GraphPad, v9.2.0). Briefly, to identify cells in G2 phase, thresholds were applied to isolate the cells that contained high CENPF intensity, whilst being negative for EdU. Intensity measurements of CMG factors (MCM7 or CDC45) on chromatin in G2 phase were taken from cells matching the established threshold intensities only. The dot plots data were then subject to normality testing through qqplots to determine the appropriate statistical testing method (non-parametric or parametric). If non-parametric, a Mann-Whitney test was performed on the mean values of the three repeats using Prism. If parametric, an un-paired t-test was performed on the mean values of the three repeats using Prism.

In order to quantify the proportion of G2 cells positive for the replisome factor: a positive signal threshold was determined by the highest intensity value in the Control sample excluding outliers (determined with Prism). Statistical significance was measured by two-tailed paired t-test. However, in some experiments this approach did not reflect the differences between samples well and instead “higher CDC45/MCM7 signal” cells were quantified as ones with signal above the median value of the control cells. Statistical significance was measured by two-tailed paired t-test.

For QIBC analysis: the captured images were processed and analyzed using HCS Studio Cell Analysis software (Fisher Scientific). For each image channel, an automated dynamic background adjustment was implemented. This adjustment was consistent across all experimental treatments. To identify individual cell nuclei, a mask based on intensity thresholds was created using the DAPI signal. This mask facilitated the analysis of pixel intensities across various channels for each nucleus. Subsequently, a table containing the data was exported for further analysis using Spotfire software (Tibco, v10.5.0.72). In each experiment, cell counts were kept consistent across different conditions for accurate comparisons. Statistical analysis was performed as above for immunofluorescence microscopy.

For cell proliferation assays analysis: statistical significance was measured by two-tailed t-test of unequal variance.

For colony assays analysis: colonies were counted and first normalised to the seeding density to obtain the percentage colony forming units. Per condition, each respective % colony forming units calculated were then normalised to untreated controls (Tet-OsTIR1 untreated) to allow for comparison between cell lines and seeding densities. Data were analyzed using one-way ANOVA, with any statistical differences detected subsequently explored using post-hoc testing (TukeysHSD) using RStudio. Colony assay data was graphed using RStudio, with the packages ggplot and ggpubr.

For quantification of western blots: 8-bit TIFF or JPEG images were analyzed, and pixel intensities determined using ImageJ. The uncalibrated OD function was selected, bands highlighted.

### Additional resources

Not applicable.

## References

[1] Hanahan D., Weinberg R.A. (2011). Hallmarks of cancer: the next generation. Cell.

[2] Riera A., Barbon M., Noguchi Y., Reuter L.M., Schneider S., Speck C. (2017). From structure to mechanism-understanding initiation of DNA replication. Genes Dev..

[3] Maric M., Maculins T., De Piccoli G., Labib K. (2014). Cdc48 and a ubiquitin ligase drive disassembly of the CMG helicase at the end of DNA replication. Science.

[4] Moreno S.P., Bailey R., Campion N., Herron S., Gambus A. (2014). Polyubiquitylation drives replisome disassembly at the termination of DNA replication. Science.

[5] Deegan T.D., Mukherjee P.P., Fujisawa R., Polo Rivera C., Labib K. (2020). CMG helicase disassembly is controlled by replication fork DNA, replisome components and a ubiquitin threshold. Elife.

[6] Deng L., Wu R.A., Sonneville R., Kochenova O.V., Labib K., Pellman D., Walter J.C. (2019). Mitotic CDK Promotes Replisome Disassembly, Fork Breakage, and Complex DNA Rearrangements. Mol. Cell.

[7] Maric M., Mukherjee P., Tatham M.H., Hay R., Labib K. (2017). Ufd1-Npl4 Recruit Cdc48 for Disassembly of Ubiquitylated CMG Helicase at the End of Chromosome Replication. Cell Rep..

[8] Sonneville R., Bhowmick R., Hoffmann S., Mailand N., Hickson I.D., Labib K. (2019). TRAIP drives replisome disassembly and mitotic DNA repair synthesis at sites of incomplete DNA replication. Elife.

[9] Sonneville R., Moreno S.P., Knebel A., Johnson C., Hastie C.J., Gartner A., Gambus A., Labib K. (2017). CUL-2LRR-1 and UBXN-3 drive replisome disassembly during DNA replication termination and mitosis. Nat. Cell Biol..

[10] Villa F., Fujisawa R., Ainsworth J., Nishimura K., Lie-A-Ling M., Lacaud G., Labib K.P. (2021). CUL2(LRR1) , TRAIP and p97 control CMG helicase disassembly in the mammalian cell cycle. EMBO Rep..

[11] Priego Moreno S., Jones R.M., Poovathumkadavil D., Scaramuzza S., Gambus A. (2019). Mitotic replisome disassembly depends on TRAIP ubiquitin ligase activity. Life Sci. Alliance.

[12] Tarcan Z., Poovathumkadavil D., Skagia A., Gambus A. (2022). The p97 segregase cofactor Ubxn7 facilitates replisome disassembly during S phase. J. Biol. Chem..

[13] Dewar J.M., Budzowska M., Walter J.C. (2015). The mechanism of DNA replication termination in vertebrates. Nature.

[14] Dewar J.M., Low E., Mann M., Räschle M., Walter J.C. (2017). CRL2Lrr1 promotes unloading of the vertebrate replisome from chromatin during replication termination. Genes Dev..

[15] Kochenova O.V., Mukkavalli S., Raman M., Walter J.C. (2022). Cooperative assembly of p97 complexes involved in replication termination. Nat. Commun..

[16] Fan Y., Koberlin M.S., Ratnayeke N., Liu C., Deshpande M., Gerhardt J., Meyer T. (2021). LRR1-mediated replisome disassembly promotes DNA replication by recycling replisome components. J. Cell Biol..

[17] Jenkyn-Bedford M., Jones M.L., Baris Y., Labib K.P.M., Cannone G., Yeeles J.T.P., Deegan T.D. (2021). A conserved mechanism for regulating replisome disassembly in eukaryotes. Nature.

[18] Low E., Chistol G., Zaher M.S., Kochenova O.V., Walter J.C. (2020). The DNA replication fork suppresses CMG unloading from chromatin before termination. Genes Dev..

[19] Wu R.A., Semlow D.R., Kamimae-Lanning A.N., Kochenova O.V., Chistol G., Hodskinson M.R., Amunugama R., Sparks J.L., Wang M., Deng L. (2019). TRAIP is a master regulator of DNA interstrand crosslink repair. Nature.

[20] Davidson I.F., Li A., Blow J.J. (2006). Deregulated replication licensing causes DNA fragmentation consistent with head-to-tail fork collision. Mol. Cell.

[21] Lin J.J., Milhollen M.A., Smith P.G., Narayanan U., Dutta A. (2010). NEDD8-targeting drug MLN4924 elicits DNA rereplication by stabilizing Cdt1 in S phase, triggering checkpoint activation, apoptosis, and senescence in cancer cells. Cancer Res..

[22] Blakemore D., Vilaplana-Lopera N., Almaghrabi R., Gonzalez E., Moya M., Ward C., Murphy G., Gambus A., Petermann E., Stewart G.S., García P. (2021). MYBL2 and ATM suppress replication stress in pluripotent stem cells. EMBO Rep..

[23] Starostina N.G., Simpliciano J.M., McGuirk M.A., Kipreos E.T. (2010). CRL2(LRR-1) targets a CDK inhibitor for cell cycle control in C. elegans and actin-based motility regulation in human cells. Dev. Cell.

[24] Sedlackova H., Rask M.B., Gupta R., Choudhary C., Somyajit K., Lukas J. (2020). Equilibrium between nascent and parental MCM proteins protects replicating genomes. Nature.

[25] Kuipers M.A., Stasevich T.J., Sasaki T., Wilson K.A., Hazelwood K.L., McNally J.G., Davidson M.W., Gilbert D.M. (2011). Highly stable loading of Mcm proteins onto chromatin in living cells requires replication to unload. J. Cell Biol..

[26] Rattner J.B., Rao A., Fritzler M.J., Valencia D.W., Yen T.J. (1993). CENP-F is a .ca 400 kDa kinetochore protein that exhibits a cell-cycle dependent localization. Cell Motil Cytoskeleton.

[27] Prigent C., Dimitrov S. (2003). Phosphorylation of serine 10 in histone H3, what for?. J. Cell Sci..

[28] Kuhne C., Banks L. (1998). E3-ubiquitin ligase/E6-AP links multicopy maintenance protein 7 to the ubiquitination pathway by a novel motif, the L2G box. J. Biol. Chem..

[29] Schneider C.A., Rasband W.S., Eliceiri K.W. (2012). NIH Image to ImageJ: 25 years of image analysis. Nat. Methods.

[30] Soucy T.A., Smith P.G., Milhollen M.A., Berger A.J., Gavin J.M., Adhikari S., Brownell J.E., Burke K.E., Cardin D.P., Critchley S. (2009). An inhibitor of NEDD8-activating enzyme as a new approach to treat cancer. Nature.

[31] Zhong W., Feng H., Santiago F.E., Kipreos E.T. (2003). CUL-4 ubiquitin ligase maintains genome stability by restraining DNA-replication licensing. Nature.

[32] Singer J.D., Gurian-West M., Clurman B., Roberts J.M. (1999). Cullin-3 targets cyclin E for ubiquitination and controls S phase in mammalian cells. Genes Dev..

[33] Jones R.M., Mortusewicz O., Afzal I., Lorvellec M., García P., Helleday T., Petermann E. (2013). Increased replication initiation and conflicts with transcription underlie Cyclin E-induced replication stress. Oncogene.

[34] Maiorano D., Krasinska L., Lutzmann M., Mechali M. (2005). Recombinant Cdt1 induces rereplication of G2 nuclei in Xenopus egg extracts. Curr. Biol..

[35] Johansson P., Jeffery J., Al-Ejeh F., Schulz R.B., Callen D.F., Kumar R., Khanna K.K. (2014). SCF-FBXO31 E3 ligase targets DNA replication factor Cdt1 for proteolysis in the G2 phase of cell cycle to prevent re-replication. J. Biol. Chem..

[36] Soucy T.A., Smith P.G., Rolfe M. (2009). Targeting NEDD8-activated cullin-RING ligases for the treatment of cancer. Clin. Cancer Res..

[37] Majera D., Skrott Z., Chroma K., Merchut-Maya J.M., Mistrik M., Bartek J. (2020). Targeting the NPL4 Adaptor of p97/VCP Segregase by Disulfiram as an Emerging Cancer Vulnerability Evokes Replication Stress and DNA Damage while Silencing the ATR Pathway. Cells.

[38] Meyer H., Weihl C.C. (2014). The VCP/p97 system at a glance: connecting cellular function to disease pathogenesis. J. Cell Sci..

[39] Patil M., Pabla N., Dong Z. (2013). Checkpoint kinase 1 in DNA damage response and cell cycle regulation. Cell. Mol. Life Sci..

[40] Vaz B., Halder S., Ramadan K. (2013). Role of p97/VCP (Cdc48) in genome stability. Front. Genet..

[41] Meerang M., Ritz D., Paliwal S., Garajova Z., Bosshard M., Mailand N., Janscak P., Hübscher U., Meyer H., Ramadan K. (2011). The ubiquitin-selective segregase VCP/p97 orchestrates the response to DNA double-strand breaks. Nat. Cell Biol..

[42] Meyer H., Bug M., Bremer S. (2012). Emerging functions of the VCP/p97 AAA-ATPase in the ubiquitin system. Nat. Cell Biol..

[43] Franz A., Ackermann L., Hoppe T. (2016). Ring of Change: CDC48/p97 Drives Protein Dynamics at Chromatin. Front. Genet..

[44] Kilgas S., Singh A.N., Paillas S., Then C.K., Torrecilla I., Nicholson J., Browning L., Vendrell I., Konietzny R., Kessler B.M. (2021). p97/VCP inhibition causes excessive MRE11-dependent DNA end resection promoting cell killing after ionizing radiation. Cell Rep..

[45] Franz A., Valledor P., Ubieto-Capella P., Pilger D., Galarreta A., Lafarga V., Fernández-Llorente A., de la Vega-Barranco G., den Brave F., Hoppe T. (2021). USP7 and VCP(FAF1) define the SUMO/Ubiquitin landscape at the DNA replication fork. Cell Rep..

[46] Elbaek C.R., Petrosius V., Sorensen C.S. (2020). WEE1 kinase limits CDK activities to safeguard DNA replication and mitotic entry. Mutat. Res..

[47] Kamentsky L., Jones T.R., Fraser A., Bray M.A., Logan D.J., Madden K.L., Ljosa V., Rueden C., Eliceiri K.W., Carpenter A.E. (2011). Improved structure, function and compatibility for CellProfiler: modular high-throughput image analysis software. Bioinformatics.

[48] Carpenter A.E., Jones T.R., Lamprecht M.R., Clarke C., Kang I.H., Friman O., Guertin D.A., Chang J.H., Lindquist R.A., Moffat J. (2006). CellProfiler: image analysis software for identifying and quantifying cell phenotypes. Genome Biol..

[49] O'Gorman S., Fox D.T., Wahl G.M. (1991). Recombinase-mediated gene activation and site-specific integration in mammalian cells. Science.

[50] Scaramuzza S., Sadurni M.M., Poovathumkadavil D., Natsume T., Rojas P., Kanemaki M.T., Saponaro M., Gambus A. (2022). Ubiquitin ligase TRAIP plays an essential role during the S phase of unperturbed cell cycle in the resolution of DNA replication – transcription conflicts. bioRxiv.

[51] Chiang K., Zielinska A.E., Shaaban A.M., Sanchez-Bailon M.P., Jarrold J., Clarke T.L., Zhang J., Francis A., Jones L.J., Smith S. (2017). PRMT5 Is a Critical Regulator of Breast Cancer Stem Cell Function via Histone Methylation and FOXP1 Expression. Cell Rep..

[52] Lakkaraju A., Umapathy A., Tan L.X., Daniele L., Philp N.J., Boesze-Battaglia K., Williams D.S. (2020). The cell biology of the retinal pigment epithelium. Prog. Retin. Eye Res..

[53] Graham M.K., Principessa L., Antony L., Meeker A.K., Isaacs J.T. (2017). Low p16(INK4a) Expression in Early Passage Human Prostate Basal Epithelial Cells Enables Immortalization by Telomerase Expression Alone. Prostate.

[54] Natsume T., Kiyomitsu T., Saga Y., Kanemaki M.T. (2016). Rapid Protein Depletion in Human Cells by Auxin-Inducible Degron Tagging with Short Homology Donors. Cell Rep..

[55] Cong L., Ran F.A., Cox D., Lin S., Barretto R., Habib N., Hsu P.D., Wu X., Jiang W., Marraffini L.A., Zhang F. (2013). Multiplex genome engineering using CRISPR/Cas systems. Science.

[56] Saito Y., Kanemaki M.T. (2021). Targeted Protein Depletion Using the Auxin-Inducible Degron 2 (AID2) System. Curr. Protoc..

[57] Forment J.V., Jackson S.P. (2015). A flow cytometry-based method to simplify the analysis and quantification of protein association to chromatin in mammalian cells. Nat. Protoc..

