# Supplementary material for: Characterizing replisome disassembly in human cells
Source: iScience. 2024 Jun 12;27(7):110260. doi: 10.1016/j.isci.2024.110260 (PMC11269944; doi:10.1016/j.isci.2024.110260)
Supplement: Document S1. Figures S1–S9, Tables S1, and S2 [file mmc1.pdf]

iScience, Volume *27*

Supplemental information

Characterizing replisome disassembly in human cells

Rebecca M. Jones, Joaquin Herrero Ruiz, Shaun Scaramuzza, Sarmi Nath, Chaoyu Liu, Marta Henklewska, Toyoaki Natsume, Robert G. Bristow, Francisco Romero, Masato T. Kanemaki, and Agnieszka Gambus

Jones et al. Supplementary Figure 1

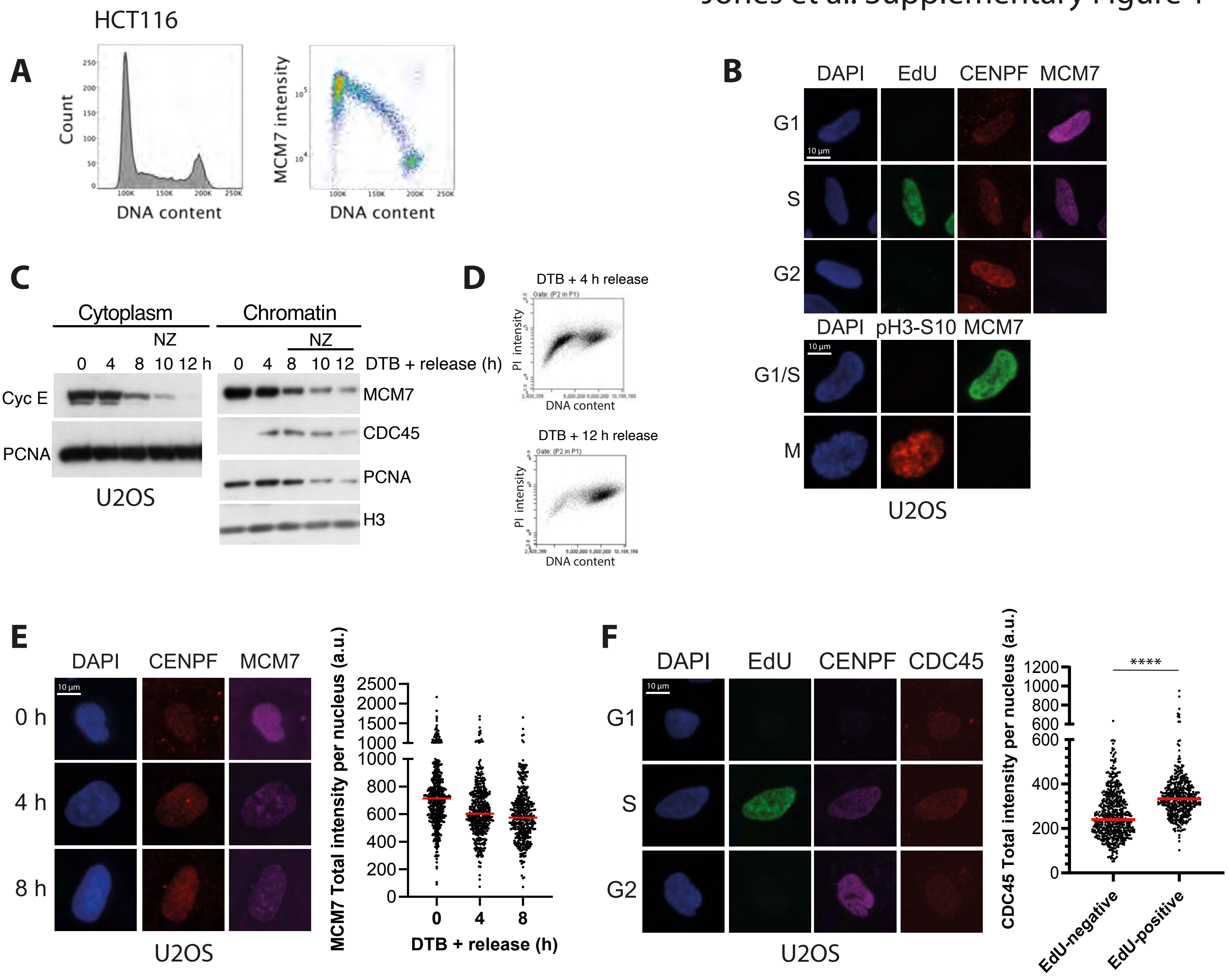

Supplementary Figure 1. Visualisation of replisome unloading from chromatin. Related to Figure 4, 5 and 6. (A) FACS analysis of CSK buffer extracted nuclei shows MCM7 loading and unloading from chromatin during the cell cycle - DNA staining and MCM7 staining. **(B)** Immunofluorescence of MCM7 chromatin binding - representative immunofluorescence images of EdU, CENPF or pH3-S10 and MCM7 binding to chromatin throughout the cell cycle. Asynchronous U2OS cells were pulse-labelled with EdU for 20 min, before cells were extracted with CSK buffer and fixed with PFA. Cells were then immunostained with indicated antibodies. **(C)** Chromatin isolation and western blotting to visualise loading and unloading of CMG factors from chromatin throughout the cell cycle. U2OS cells were synchronised with DTB and released for indicated length of time. Nocodazole was added at later timepoints to stop cells from entering the next cell cycle. Cells were extracted with CSK buffer and chromatin samples analysed through western blotting with indicated antibodies. **(D)** Cells from (C) were analysed by FACS to determine cell cycle stage. **(E)** MCM7 can be visualised unloading from chromatin by immunofluorescence in synchronised cells - representative immunofluorescence images of CENPF and MCM7 binding to chromatin in U2OS cells synchronised with DTB and released for indicated length of time. MCM7 intensity was quantified (>380 cells/sample). Red lines indicate the median (n=1). **(F)** CDC45 can be visualised loading and unloading from chromatin by immunofluorescence - representative immunofluorescence images of EdU, CENPF and CDC45 binding with chromatin at different stages of the cell cycle in asynchronous U2OS cells. CDC45 intensity was quantified in EdU-negative vs EdU-positive cells (AVG >150 cells/sample). Red lines indicate the median (n=3) ($p=<0.0001$, Two-tailed Mann Whitney test).

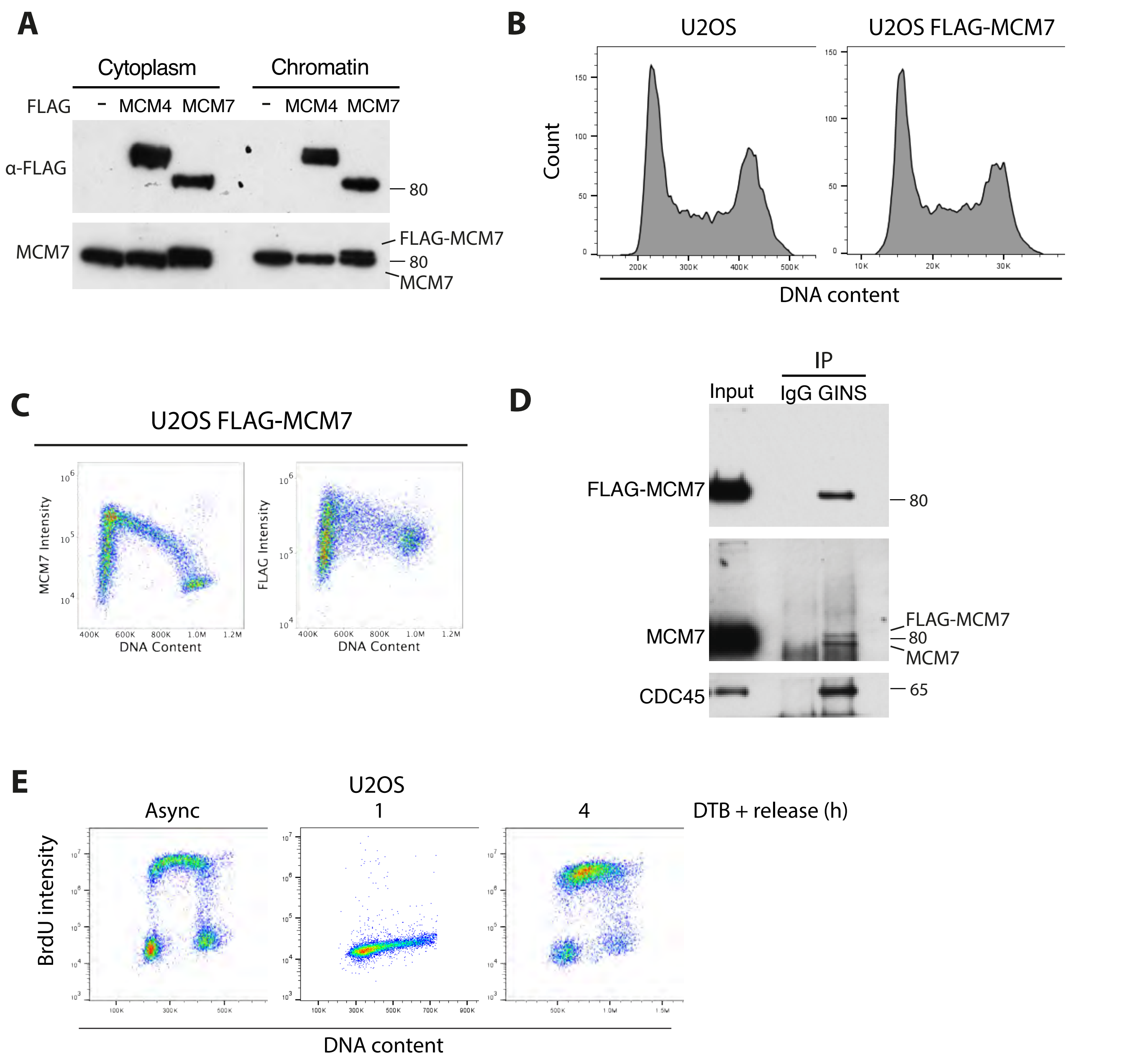

Supplementary Figure 2. Tagging MCM7 with FLAG-tag does not affect its function. Related to Figure 2. (A) Cytoplasmic or chromatin fractions were extracted from U2OS cells expressing FLAG-MCM4 or FLAG-MCM7 and samples analysed by western blotting with indicated antibodies. **(B)** Cell cycle profiles for U2OS and FLAG-MCM7-expressing U2OS cells. **(C)** MCM7 chromatin binding pattern of U2OS cells expressing FLAG-MCM7. Representative data shown are FACS plots from the same sample, with MCM7 and FLAG intensity measured in different channels. **(D)** U2OS cells expressing FLAG-MCM7 were synchronised in S-phase with DTB, with 4 h release and GINS was immunoprecipitated from chromatin lysates. **(E)** U2OS cells synchronised with DTB, released for indicated time points and pulsed with BrdU for 1 hour prior to harvesting. BrdU intensity and DNA content measured in each sample by FACS.

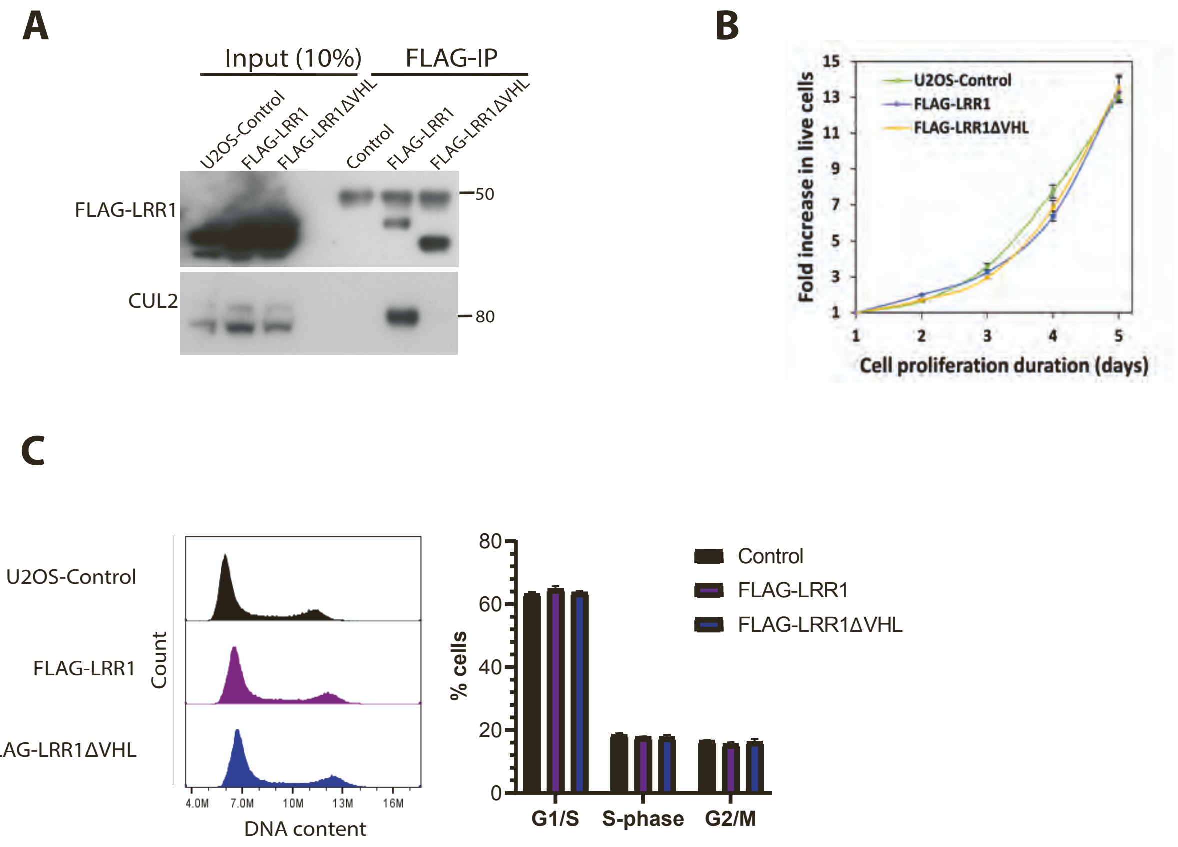

Supplementary Figure 3. Expression of FLAG-LRR1 or FLAG-LRR1ΔVHL does not affect cell proliferation. Related to figure 3. (A) FLAG-LRR1 or FLAG-LRR1ΔVHL were immunoprecipitated from whole cell extracts of U2OS cells with FLAG M2 beads and analysed by western blotting with the indicated antibodies. **(B)** Fold increase of live U2OS cells expressing FLAG-LRR1 or FLAG-LRR1ΔVHL (n=3); error bar indicates SD. **(C)** Analysis of the cell cycle profiles of control U2OS cells, or U2OS cells transiently transfected with FLAG-LRR1 or FLAG-LRR1ΔVHL plasmids for 48 h - representative histograms and quantification of average percentage of cells in G1, S and G2/M phase, with SD (n=3) (all p=ns).

Jones et al Supplementary Figure 4

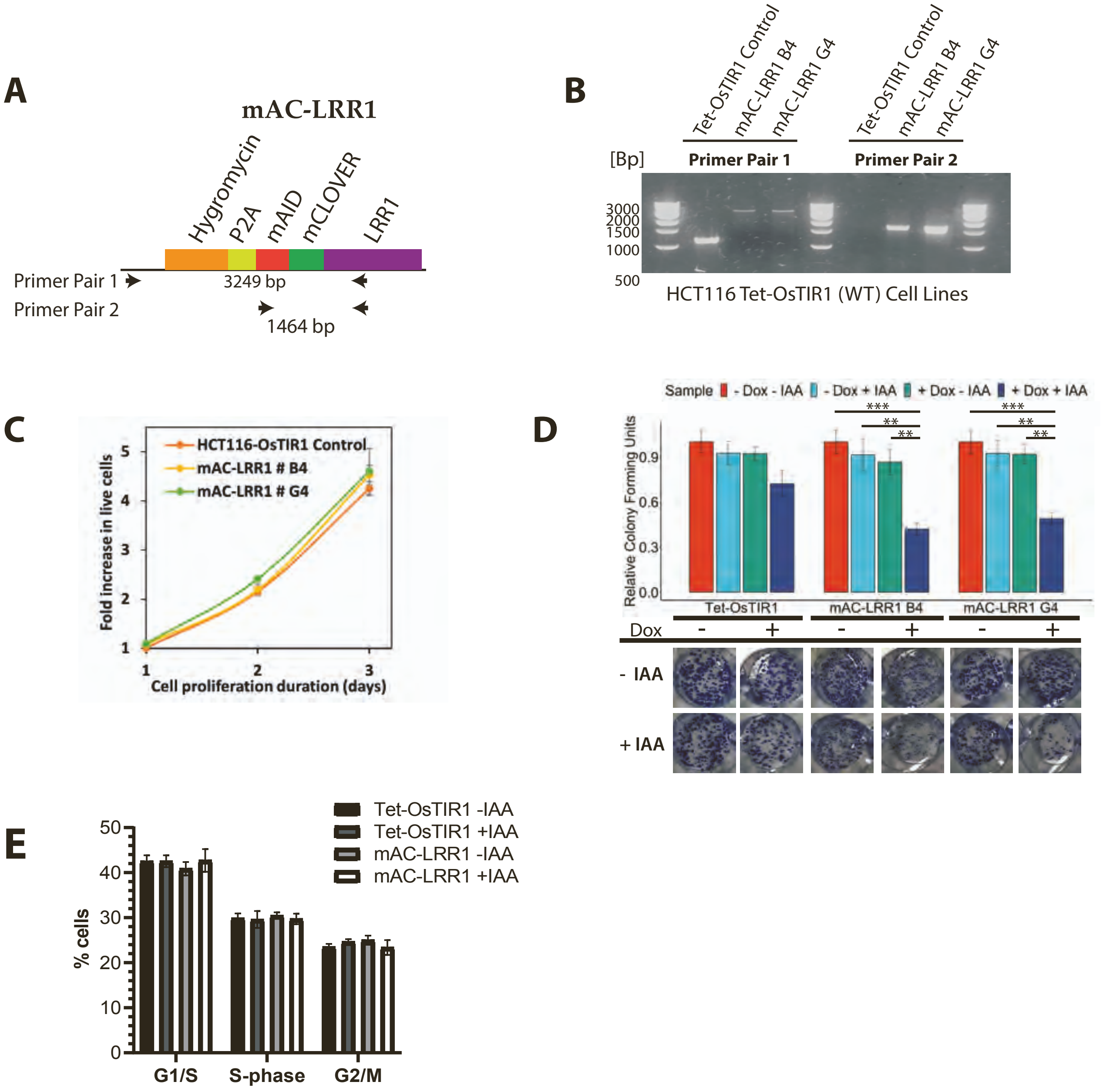

Supplementary Figure 4. Characterisation of HCT116 cells with auxin (IAA)-induced LRR1 degron. Related to Figure 3 and 5. (A) Tagging layout for mAC-LRR1 degron cells. Endogenous LRR1 was tagged N-terminally with a degron tag, p2a self-cleavage site, and hygromycin resistance marker. Arrows indicate the position of primers deigned to screen for bi-allelic gene tagging. Expected sizes resulting from the amplification of these regions is also shown. **(B)** Example DNA agarose gel following genomic PCR to screen for cells in HCT116 Tet-OsTIR1 (WT) background. Shown are the confirmed bi-allelic cell lines utilised for this study. **(C)** Fold increase in numbers of live HCT116 cells expressing mAC-LRR1, monitored for 3 days (n=3); error bars indicate SD. **(D)** Colony assays and quantification showing cell viability for the selected HCT116 mAC-LRR1 cells. Colonies were counted (n=3) and normalised to untreated controls. One-way ANOVA: Tet-OsTir1 (p=0.0749); mAC-LRR1 B4 (p=0.000274); mAC-LRR1 G4 , (p=0.000154). Pairwise hypothesis testing within the degron cells (Tukeys HSD), with significant differences shown on the plot. **(E)** Quantification for cell cycle profile of HCT116 cells expressing mAC-LRR1 treated with Tetracycline (1 µg/ml) and IAA (100 µM) for 24 h (n=4); mean value with SEM (all p=ns).

Jones et al Supplementary Figure 5

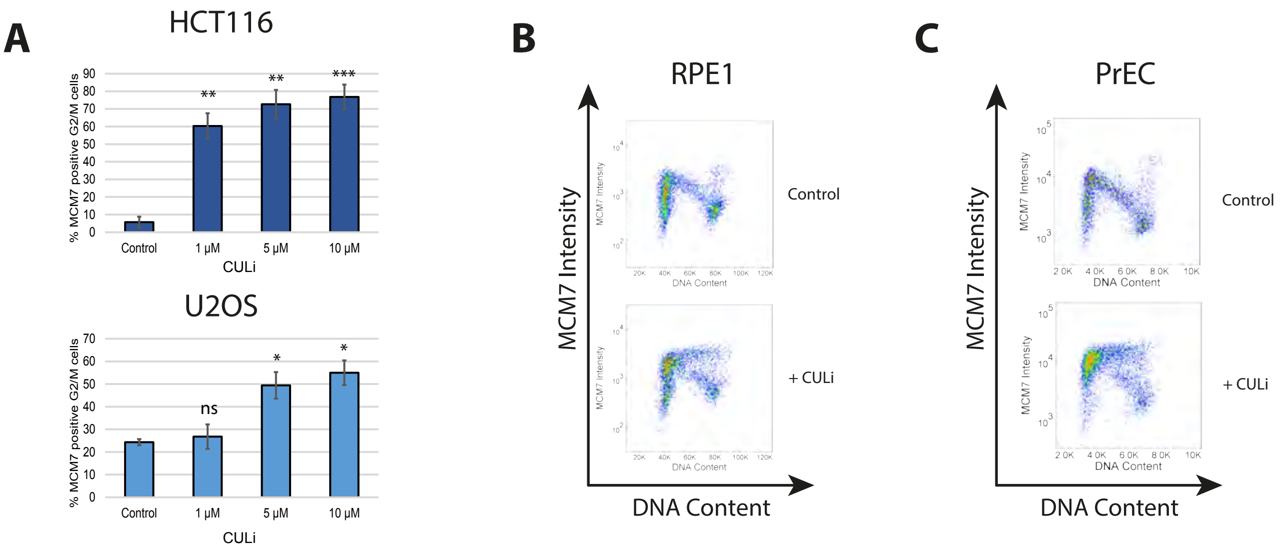

Supplementary Figure 5. Cullin activity inhibition causes replisome retention on chromatin. Related to Figure 4. (A) FACS analysis quantification of % of G2/M cells positive for MCM7 from asynchronous HCT116 and U2OS cells, treated with differing concentrations of CULi for 6 hours (n=3). Each data set compared with the Control. HCT116: 1 µM (p=0.00199), 5 µM (p=0.0019), 10 µM (p=0.00085). U2OS: 5 µM (p=0.0261), 10 µM (p=0.0372). All Two-tailed paired t-tests.**(B)** Example FACS plots for the total MCM7 intensity (y axis) against DNA content (x axis) as in Figure 4A but for RPE1 cells. **(C)** Same as (B) but for PreC cells.

Jones et al Supplementary Figure 6

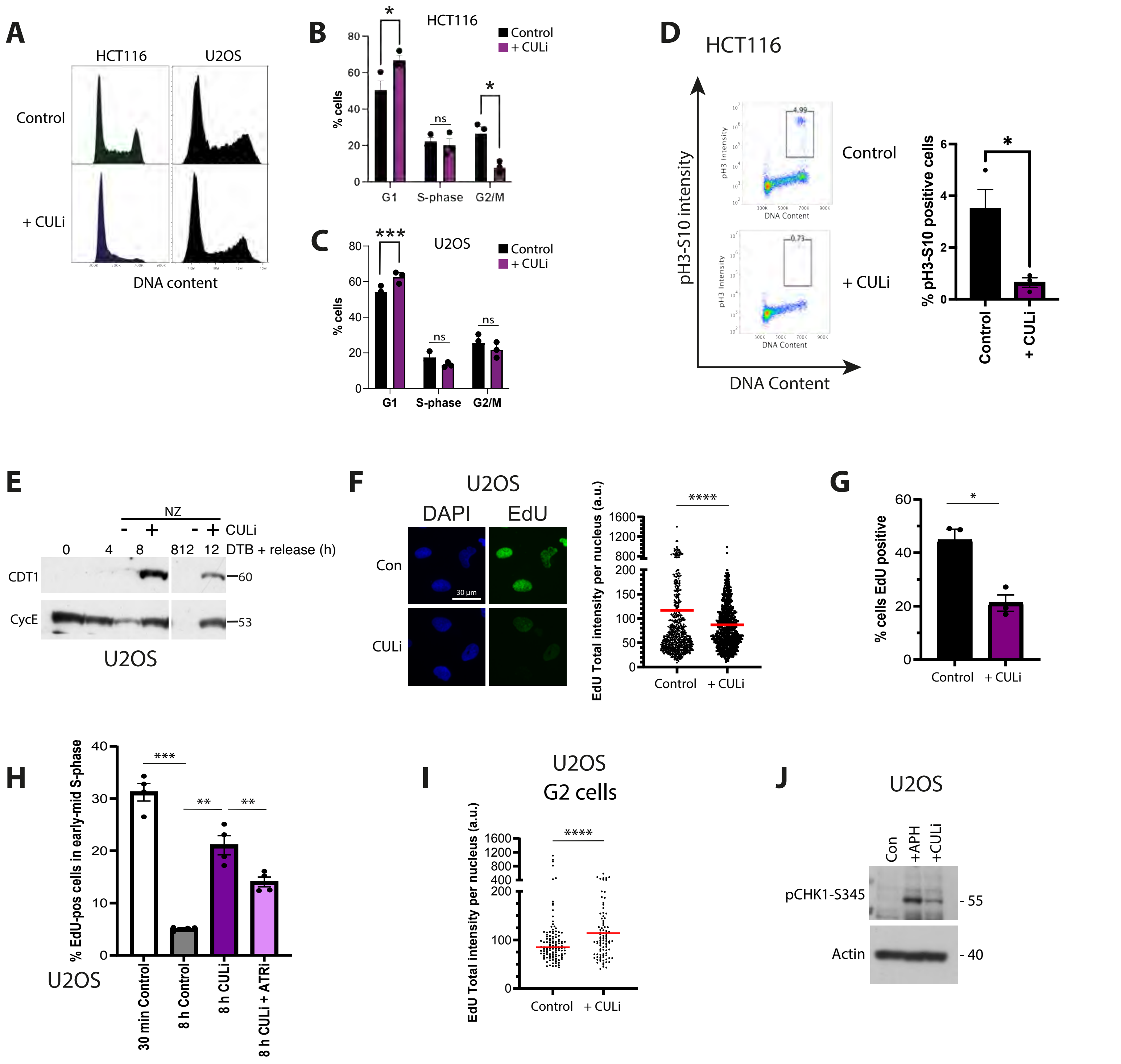

Supplementary Figure 6. The effects of cullin activity inhibition. Related to Figure 4. **(A)** CULi affects cells progression through S-phase. Cell cycle profiles of asynchronous HCT116 and U2OS cell lines following optional CULi treatment for 6 h - representative histograms depicting total DNA content (x axis). **(B)** Quantification of the proportion of cells in G1 (2N DNA content), S-phase (2N < DNA Content > 4N), or G2/M (4N DNA content) for HCT116 cells from (A) (n=3): G1: p=0.0434; G2/M: p=0.0134; mean value with SEM **(C)** Same as (B) but for U2OS cells: G1: p=0.0004. **(D)** Analysis of the propotion of mitotic (pH3-S10-positive) HCT116 cells following 6 hours CULi treatment - example FACS plots and quantification showing pH3-S10 intensity (y axis) against total DNA content (x axis) (n=3) (p=0.0212); mean value with SEM. **(E)** CULi causes an accumulation of CDT1 and Cyclin E. U2OS cells were synchronised with DTB and released for indicated time points ±CULi ±nocodazole (NZ). Chromatin samples analysed through western blotting with indicated antibodies. **(F)** CULi affects cells' ability to synthesise DNA - representative immuno-fluorescence images and quantification of EdU in asynchronous U2OS cells, treated ±CULi for 6 h (n=3). Red lines indicate the median (p=<0.0001, Two-tailed Mann Whitney test). **(G)** Quantification of % cells positive for EdU from (E) (n=3); mean value with SEM (p=0.0103, Two-tailed paired t-test). **(H)** CULi delays S-phase progression. U2OS cells pulsed with EdU for 30 min and released for 8 h ±CULi ±ATRi before cells were harvested for EdU and PI FACS analysis (n=3); mean value with SEM; 8 h control vs 30 min control (p=0.0007); 8 h CULi vs 8 h Control (p=0.0039); 8 h CULi +ATRi vs 8 h CULi (p=0.0046). All Two-tailed paired t-tests. **(I)** Quantification of EdU total intensity in G2 cells from Figure 4I (n=3). Red lines indicate the median (p=<0.0001, Two-tailed Mann-Whitney test). **(J)** CULi causes an accumulation of pCHK1-S345. Asynchronous U2OS cells were treated ±CULi for 6 h. Nucleoplasmic samples analysed through western blotting with indicated antibodies.

Jones et al Supplementary Figure 7

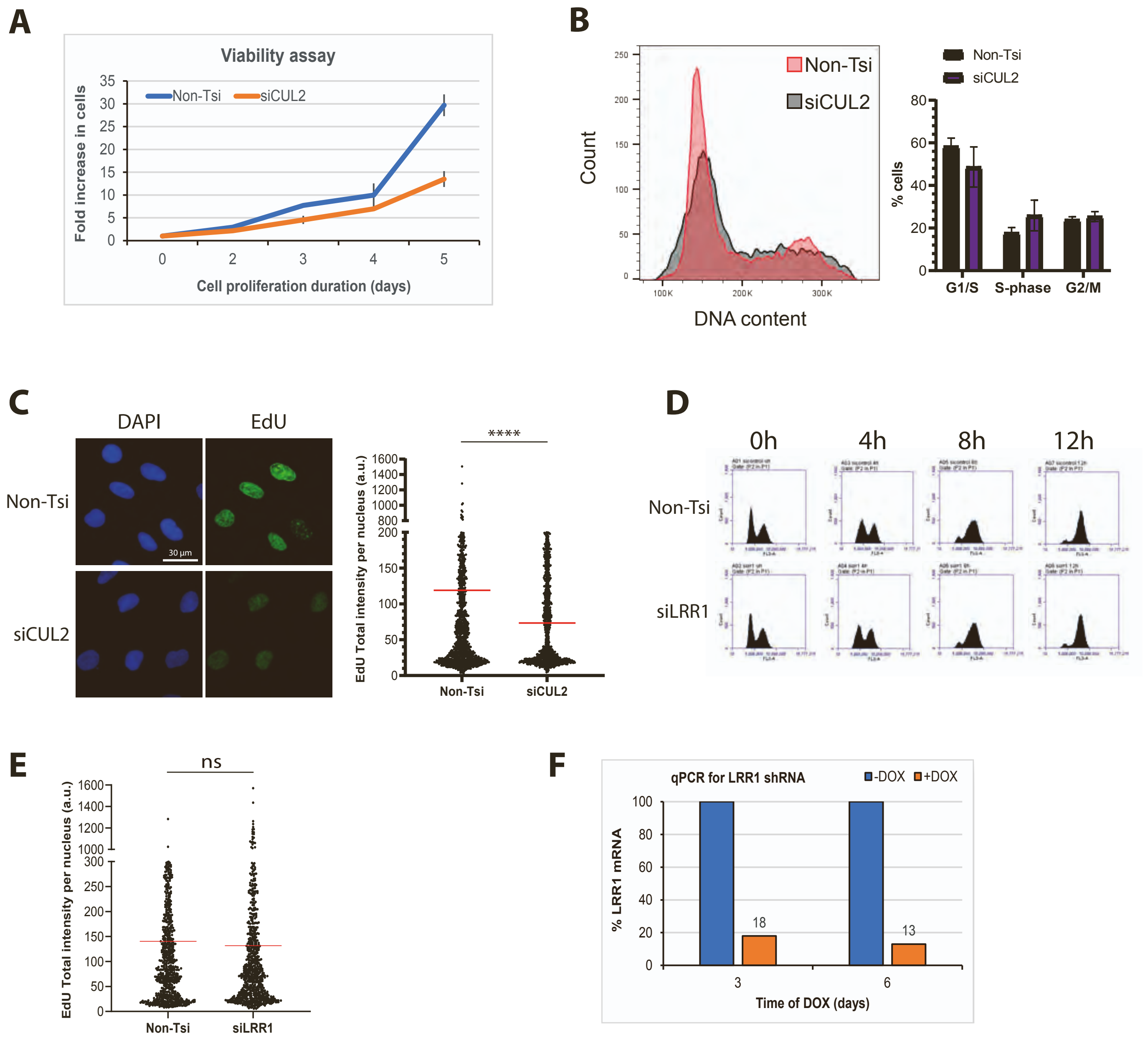

Supplementary Figure 7. Phenotypes following downregulation of CUL2 or LRR1. Related to Figure 5. (A) Growth assay to compare proliferation of U2OS cells with Non-T or CUL2 siRNA. Cells were transfected on day 1 (n=2). **(B)** Cell cycle profile of U2OS cells depleted of CUL2 with siRNA for 3 days with quantification (n=2); mean with SEM. **(C)** CUL2 downregulation reduces EdU incorporation - representative images of EdU-positive U2OS cells from asynchronous population following transfection with Non-T or CUL2 siRNA for 3 days and quantification of total EdU intensities (n=3). Red lines indicate the median (p=<0.0001, Two-tailed Mann-Whitney test). **(D)** LRR1 downregulation does not affect cell cycle progression. U2OS cells depleted of LRR1 with siRNA were synchronised with DTB and released for indicated time points and cell cycle profiles analysed by FACS (n=1). **(E)** LRR1 downregulation does not reduce EdU incorporation. Quantification of total EdU intensities in asynchronous population of U2OS cells following transfection with Non-T or LRR1 siRNA for 3 days (n=2). Red lines indicate the median. **(F)** qPCR analysis for LRR1 mRNA in HEK293T cells stably expressing DOX-inducible LRR1 shRNA for 3 or 6 days (n=1).

Jones et al. Supplementary Figure 8

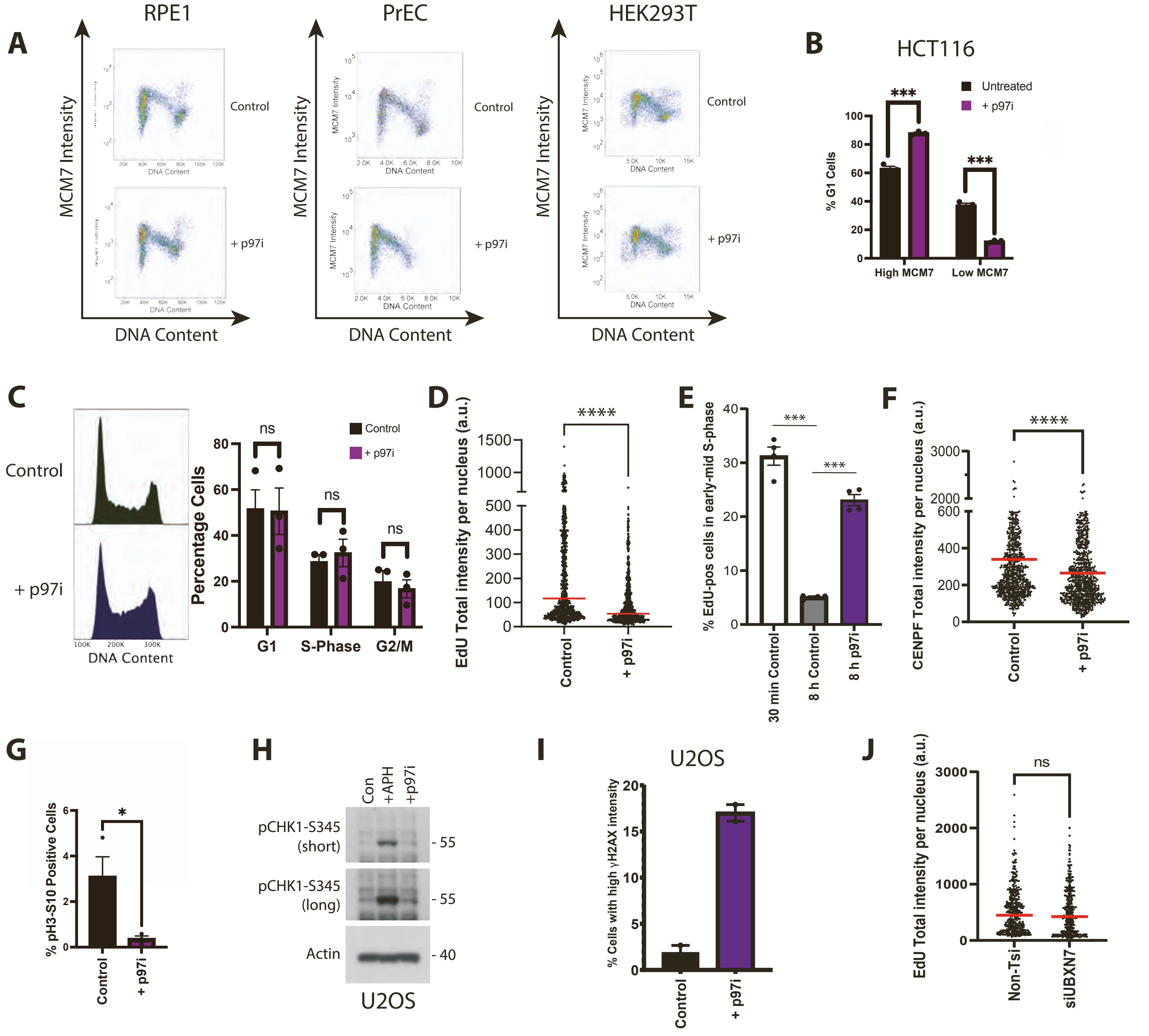

Supplementary Figure 8. The effects of p97 activity inhibition. Related to Figure 6. (A) Example FACS plots for the total MCM7 intensity (y axis) against DNA content (x axis) as in Figure 6A but for RPE1, PrEC and HEK293T cells. **(B)** Quantification of percentage of G1 cells positive for MCM7 in HCT116 cells from Figure 6A (High MCM7: p=0.000196; Low MCM7: p=0.000171). **(C)** Analysis of the cell cycle profiles in asynchronous HCT116 cells following p97i treatment - representative FACS plots depicting histograms of total DNA content and quantification of the proportions of cells in each cell cycle stage (n=3); mean values with SEM. **(D)** Quantification of total EdU intensity in U2OS cells treated ±p97i for 6 hours from Figure 6C (n=3). Red lines indicate the median (p=<0.0001, Two-tailed Mann-Whitney test). **(E)** p97i delays S-phase progression. U2OS cells pulsed with EdU for 30 min and released for 8 h ±p97i before cells were harvested for EdU and PI FACS analysis (n=3); mean value with SEM. 8 h Control vs 30 min Control (p=0.0007); 8 h p97i vs 8 h Control (p=0.0006). Both Two-tailed paired t-tests. **(F)** Quantification of total CENPF intensity in U2OS cells treated ±p97i for 6 hours from Figure 6C (n=3). Red lines indicate the median (p=<0.0001, Two-tailed Mann-Whitney test). **(G)** Quantification of the total proportions of cells positive for pH3-S10 (p=0.0369); mean values with SEM. **(H)** Activation of pCHK1-S345 with p97i treatment. Asynchronous U2OS cells were treated with DMSO, aphidicolin (APH) or p97i for 6 h before nucleoplasmic samples were analysed by western blotting with indicated antibodies. **(I)** γH2AX signalling with p97i treatment. Asynchronous U2OS cells were treated with p97i for 6 h before cells were fixed with PFA and immunostained for γH2AX-S10. Shown is the quantification of % cells with high γH2AX intensity (n=2); mean value with SEM (Con AVG >450 cells; p97 AVG >340 cells). **(J)** Quantification of total EdU intensity in all U2OS cells treated with Non-T or UBXN7 siRNA for 72 hours (n=2). Red lines indicate the median.

A

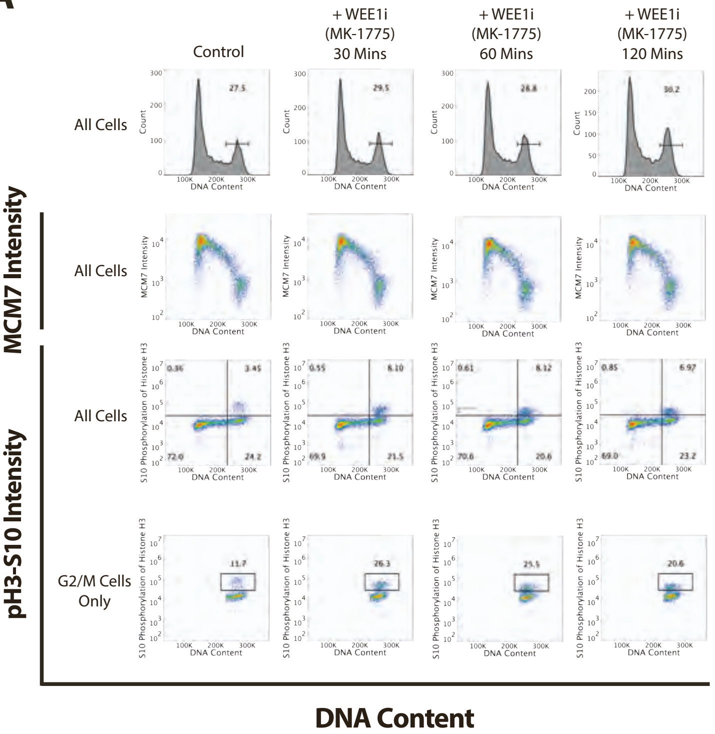

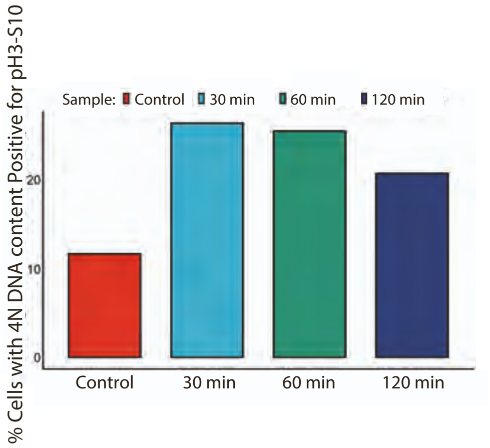

B

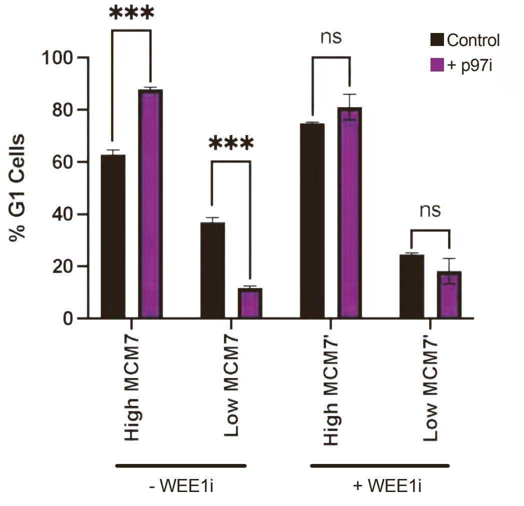

Supplementary Figure 9. Inhibition of WEE1 kinase allows CULi or p97i treated cells to enter mitosis. Related to Figure 7. (A) Validation of the ability of WEE1i to push cells into mitosis. Asynchronous HCT116 cells were treated for the indicated time with WEE1i - representative FACS plots showing the overall cell cycle, MCM7 binding pattern, pH3-S10 staining pattern, and mitotic cells and quantification of the proportion of mitotic cells (4N DNA cells positive for pH3-S10). **(B)** Effects of p97i in combination with WEE1i on cell cycle progression. Cells treated as in Fig 7G. Quantification of the approximate amounts of cells undergoing origin licensing.

Supplementary tables

Table S1. Oligonucleotides for depletion of LRR1 with shRNA. Related to Supplementary Figure 7F and Figure 5L.

REAGENT or RESOURCE	SOURCE	IDENTIFIER
**Oligonucleotides**		
TRIPZ Lentiviral Inducible LRR1 shRNA - 1 ATGAGAGCCATATGGAATC (5'- 3')	Horizon Discovery	Clone Id: V2THS_25090
TRIPZ Lentiviral Inducible LRR1 shRNA - 2 CAAACACAAATTTTTGCGG (5'- 3')	Horizon Discovery	Clone Id: V3THS_339461
TRIPZ Lentiviral Inducible LRR1 shRNA - 3 CTCCCTTAGCTCATAGCGG (5'- 3')	Horizon Discovery	Clone Id: V3THS_339460
TRIPZ Lentiviral Inducible LRR1 shRNA – 4 TCTCCCTTAGCTCATAGCG (5'- 3')	Horizon Discovery	Clone Id: V3THS_339458
TRIPZ Lentiviral Inducible LRR1 shRNA – 5 TTTGCGGTATCCAAATCTT (5'- 3')	Horizon Discovery	Clone Id: V3THS_339456
TRIPZ Lentiviral Inducible LRR1 shRNA - 6 TAGACAGATATCCACAGGA (5'- 3')	Horizon Discovery	Clone Id: V3THS_339457

Table S2. Antibody dilutions used. Related to all Figures except Supplementary Figure 4 and Supplementary Figure 7.

PROTEIN	ISOTYPE	SOURCE	CATALOG NO.	DILUTION
**Primary antibodies used for immunofluorescence**				
CENPF	Rabbit	Abcam	ab5	1:200
MCM7 (clone 141.2)	Mouse	Santa Cruz	Sc-9966	1:100
Mitosin (clone 11)	Mouse	Fisher Scientific	15805639	1:200
CDC45 (clone D7G6)	Rabbit	Cell Signaling Technology	11881S	1:100
pH3-S10 (clone D2C8)	Rabbit	Cell Signaling Technology	3377S	1:1000
γH2AX-S10 (clone JBW301)	Mouse	Millipore	05-636	1:1000
**Primary antibodies used for flow cytometry**				
MCM7 (clone 141.2)	Mouse	Santa Cruz	Sc-9966	1:500
BrdU (clone B44)	Mouse	BD Biosciences	347580	1:5
FLAG (clone M2)	Mouse	Sigma	F3165	1:1000
**Secondary antibodies for immunofluorescence and FACS**				
Anti-mouse Alexa Fluor 488	Goat	Invitrogen	A-32723	1:500
Anti-mouse Alexa Fluor 647	Goat	Invitrogen	A-21235	1:500
Anti-rabbit Alexa Fluor 555	Goat	Invitrogen	A-21428	1:500
**Primary antibodies for western blotting**				

Cyclin E (clone E-4)	Mouse	Santa Cruz	Sc-377100	1:5000
PCNA	Mouse	Sigma	P8825	1:1000
MCM7 (clone 141.2)	Mouse	Santa Cruz	Sc-9966	1:100
MCM7 (clone H-300)	Rabbit	Santa Cruz	Sc-22782	1:1000
CDC45 (clone G-12)	Mouse	Santa Cruz	Sc-55569	1:500
CDC45	Rat	Heinz Peter Nasheuer		1:500
CDC45 (clone D7G6)	Rabbit	Cell Signaling Technology	11881S	1:500
Histone H3	Rabbit	Cell Signaling Technology	9715S	1:2000
K48-linked ubiquitin (clone D9D5)	Rabbit	Cell Signaling Technology	12805S	1:1000
FLAG (clone M2)	Mouse	Sigma	F3165	1:1000
GINS	Sheep	Raised in-house against purified human GINS protein		1:1000
CUL2 (clone EPR3104(2))	Rabbit	Abcam	166917	1:2000
MCM2 (clone 46/BM28)	Mouse	BD Biosciences	610700	1:2000
Tubulin (clone DM1A)	Mouse	Sigma	T9026	1:1000
GFP (clone 3H9)	Rat	Chromotek	3H9	1:500
LRR1	Rabbit	Atlas	HPA069364	1:500
CDT1 (clone H-300)	Rabbit	Santa Cruz	Sc-28262	1:1000
Actin (clone C4)	Mouse	Santa Cruz	Sc-4777	1:5000
p-CHK1-S345	Rabbit	Cell Signaling Technology	2341S	1:500
**Secondary antibodies for western blotting**				
Mouse	-	Sigma	A5278	1:5000
Rabbit	-	Sigma	A9161	1:25000
Sheep	-	Sigma	A3415	1:10000
Rat	-	Sigma	A9542	1:5000
